# Associations of Individual- and Neighborhood-Level Factors with Racial and Ethnic Disparities in Timely Dementia Diagnosis

**DOI:** 10.1007/s11606-026-10420-8

**Published:** 2026-05-01

**Authors:** Yuting Qian, Fan Li, Xi Chen

**Affiliations:** 1 Department of Health Policy and Management, Yale University, New Haven, CT USA; 2 Department of Biostatistics, Yale University, New Haven, CT USA; 3 Department of Economics, Yale University, New Haven, CT USA; 4 National Bureau of Economic Research, Cambridge, MA USA; 5 Department of Health Policy and Management, Department of Economics, Yale University, NBER, IZA, New Haven, CT USA

**Keywords:** Timely dementia diagnosis, Disparities, Education, Neighborhood socioeconomic factors, Health care access

## Abstract

**Background:**

Underdiagnosis of dementia disproportionately affects racial and ethnic minority groups. As disease-modifying treatments and advances in dementia care models emerge, it is critical to ensure equitable, timely detection of dementia among older adults.

**Objective:**

To examine racial and ethnic disparities in the timely diagnosis of dementia and determine what individual- and neighborhood-level factors explain most of these disparities.

**Design:**

Retrospective observational study using 1998–2020 Medicare-linked survey data.

**Participants:**

U.S. adults aged ≥ 65 classified as having dementia in the Health and Retirement Study (HRS), linked to Medicare claims.

**Main Measures:**

Timely diagnosis of dementia, defined as a clinical diagnosis of dementia recorded in Medicare claims within 3 years before or 1 year after the month of the HRS interview at which dementia was first observed, was compared with delayed or missed dementia diagnosis.

**Key Results:**

In this population-based study of 3, 429 U.S. older adults classified as having dementia in the HRS, non-Hispanic Black (odds ratio, 0.71 [95% CI, 0.57 to 0.89]) and Hispanic individuals (odds ratio, 0.61 [95% CI, 0.44 to 0.85]) were less likely than non-Hispanic White individuals to receive a timely diagnosis after adjusting for non-modifiable demographics and health conditions. Educational attainment explained the largest share of the disparities (48.4% for non-Hispanic Black individuals and 61.9% for Hispanic individuals). Neighborhood affluence accounted for 27.3% and 17.8%, respectively, followed by densities of health practitioner offices (15.8% and 14.6%), physician offices (9.6% and 12.4%), and dementia specialist evaluation (6.6% and 5.5%).

**Conclusions:**

A large proportion of racial and ethnic disparities in timely diagnosis among older adults with dementia were explained by educational attainment, followed by neighborhood affluence, the densities of health practitioner offices, physician offices, and dementia specialist evaluation. The findings point to key directions for targeted interventions to improve dementia detection and advance equity in dementia care.

**Supplementary Information:**

The online version contains supplementary material available at https://doi.org/10.1007/s11606-026-10420-8.

## INTRODUCTION

Alzheimer’s disease and related dementias (ADRD), neurodegenerative conditions characterized by progressive cognitive decline, are a leading cause of disability and dependency among older adults and are among the most expensive diseases in the United States (U.S.).^1–3^ High-quality care for individuals with dementia begins with a timely diagnosis, a crucial first step that allows providers to identify reversible causes, optimize treatment, coordinate medical care, and help individuals and families plan for the future.^4,5^ However, up to 60% of dementia cases go undiagnosed in U.S. primary care.^6^ Although underdiagnosis is a critical public health concern across all populations, it disproportionately affects racial and ethnic minority groups, with non-Hispanic Black and Hispanic individuals less likely than non-Hispanic White individuals to receive a timely diagnosis,^7–11^ despite having a higher risk of developing dementia.^12–15^

Recent advancements in dementia care, including a new class of disease-modifying treatments for early-stage Alzheimer’s disease and the Centers for Medicare & Medicaid Services’ (CMS) adoption of an alternative payment model for comprehensive dementia care, represent a turning point for patients with dementia.^16,17^ However, racial and ethnic disparities in the timely diagnosis of dementia may hinder equitable access to these novel treatments and care models. Ensuring equitable, timely detection of dementia is therefore critical for improving patient outcomes across all communities.

Prior studies have documented racial and ethnic differences in factors conceptually linked to delayed dementia diagnosis, including fewer socioeconomic resources,^18^ lower levels of patient education and knowledge about dementia,^18–20^ and mistrust of health care providers among minority populations.^21^ Disparities in access to detection-related care have also been reported,^22^ with minority groups less likely to discuss memory concerns with clinicians,^23^ receive cognitive screening,^24,25^ or access dementia specialists.^26–28^ Separately, socioeconomic factors associated with the timeliness of dementia diagnosis in the general population,^8–10,29–31^ including neighborhood socioeconomic conditions, have also been found to contribute to racial and ethnic differences in diagnosis and treatment for other conditions.^32–34^ However, direct quantitative evidence on which factors drive racial and ethnic disparities in timely dementia diagnosis, and the magnitude of their contributions, remains limited.

A deeper understanding of these mechanisms is needed to inform interventions and advance health equity. Building on prior studies that have hypothesized socioeconomic and access-related differences as potential contributors but have not quantified their roles, this study examined the extent to which individual- and neighborhood-level factors, including sociodemographic factors and access to health care services, are associated with racial and ethnic differences in the timely diagnosis of dementia among older U.S. adults.

## METHODS

### Data Source

We used data from the 1998–2020 Health and Retirement Study (HRS), linked to Medicare claims from 1995 to 2021. The HRS is a nationally representative biennial survey of U.S. older adults with detailed information on demographics, socioeconomic status, and cognitive and physical functioning. By linking HRS data to Medicare claims, we could track both cognitive status and diagnosis records over time. Additionally, we merged the HRS-Medicare-linked data with the National Neighborhood Data Archive (NaNDA), a public archival resource containing composite index measures of sociodemographic status and health care service availability at the neighborhood level across the U.S. that has been used in recent studies.^35–37^

### Study Population

**Figure 1:**
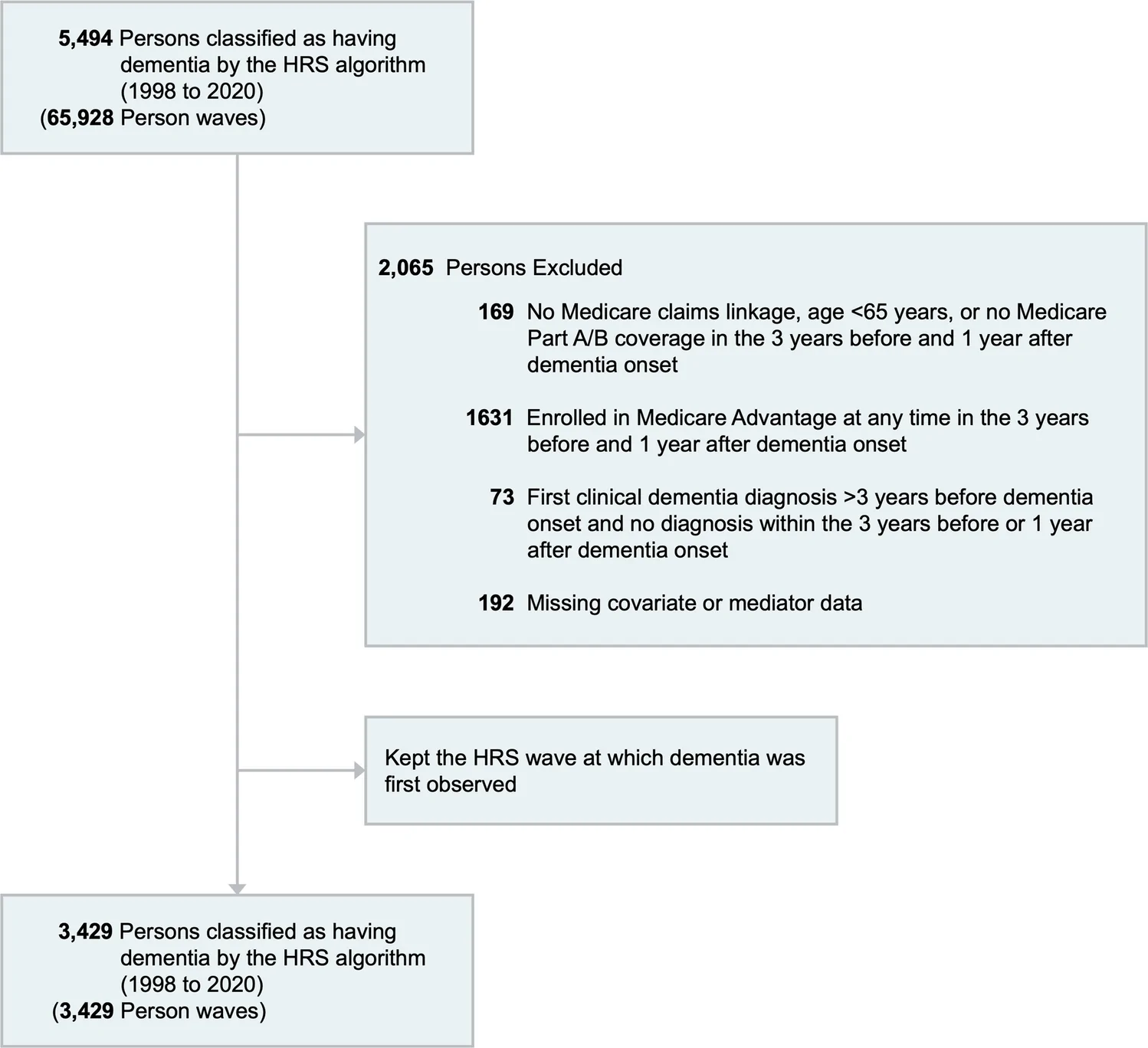
Generation of the analytic sample. Abbreviations: HRS, Health and Retirement Study. Notes: The figure illustrates the sample selection process. “HRS algorithm” refers to the predicted cognition and dementia measures developed by Hudomiet et al. (2022). Dementia onset refers to the HRS interview at which dementia was first observed.

We included HRS participants from 1998 to 2020 who were classified as having dementia in HRS (defined below), consented to Medicare records linkage, and were enrolled in Medicare fee-for-service (FFS) during the 3 years preceding and 1 year following the HRS interview at which dementia was first observed (i.e., dementia onset). This 4-year window was used to define our outcome (see the “Outcome Measures” section). We excluded beneficiaries enrolled in Medicare Advantage (MA) at any point during the 4-year window because complete claims data are not consistently available for MA enrollees. Enrollment status was ascertained from the Medicare Beneficiary Summary File. Individuals with missing covariate or mediator data (≈ 3%) were excluded (Fig. 1). Individuals with claims-based diagnoses who did not meet the HRS algorithmic criteria for dementia were not eligible for the analytic sample, as the study focuses on diagnostic timing relative to the onset of symptomatic dementia (Table S1).

We assigned dementia status to all person-wave observations using the predicted cognition and dementia measures developed by Hudomiet et al.,^38^ a validated algorithm that provides more accurate estimates of dementia prevalence by race and ethnicity and is better suited for examining racial and ethnic disparities than existing HRS-based methods.^38,39^ Hudomiet et al.’s estimates span 2000–2016; we applied their published algorithms^40^ to extend estimates to additional years (1998, 2018–2020), with replicated estimates closely aligning with the originals. Details on the dementia classification algorithm, replication, and comparisons are provided in the Online Supplement (Methods S1).

### Outcome Measures

Our primary outcome was a binary indicator of receiving a timely dementia diagnosis (versus not). Participants were categorized based on the timing of a clinical dementia diagnosis in Medicare claims relative to dementia onset as defined in the HRS, which served as a standardized, symptom-based reference point. Timely diagnosis was defined as a clinical diagnosis of dementia recorded in Medicare claims within 3 years before or 1 year after dementia onset. Individuals without a diagnosis within this 4-year window were classified as not timely diagnosed, including those whose first diagnosis occurred more than 1 year after onset (delayed diagnosis) and those who never received a diagnosis in claims through December 31, 2021, or prior to death (missed diagnosis). Those without a timely diagnosis whose first clinical dementia diagnosis preceded the 4-year window were excluded to avoid misclassification (Fig. 1).

Although no consensus exists on the operational definition of a timeliness window, our approach aligns with prior studies informed by Medicare claims-based dementia identification periods and clinical judgment.^7,41^ Dementia diagnoses were identified using the *International Classification of Diseases, Ninth or Tenth Revision, Clinical Modification* codes widely used in prior studies^9,42,43^ (Table S2).

### Race and Ethnicity

Race and ethnicity were self-reported by HRS participants using fixed categories provided by the survey instrument. For analysis, participants were classified into mutually exclusive groups: non-Hispanic White, non-Hispanic Black, Hispanic, and non-Hispanic other. Analysis focused on the first three groups due to the small sample size of the non-Hispanic other category.

### Covariates

Following the Institute of Medicine (IOM) definition of a health care disparity (a difference in health services between racial and ethnic groups not justified by differences in health status or clinical need),^44,45^ widely used in prior studies,^46–49^ we adjusted for non-modifiable demographics and health conditions to ensure comparisons of timely diagnosis were made across clinically comparable groups. These included age, gender, comorbid conditions, cognition score (algorithm-estimated latent cognition by Hudomiet et al.), and number of limitations in activities of daily living (ADLs) and instrumental activities of daily living (IADLs) (Table S3). Health conditions were measured at the wave of dementia onset, as individuals’ clinical profiles at onset are most proximate to and most likely to shape the likelihood of timely diagnosis.

### Potential Mediators

We evaluated mediators across three domains: individual-level socioeconomic status (income and educational attainment), neighborhood-level socioeconomic factors (neighborhood disadvantage and affluence), and health care access factors, including any pre-diagnosis visit to a dementia specialist and the density of physician and non-physician health practitioner offices and clinics at the neighborhood level. Dementia specialties included neurology, psychiatry, neuropsychiatry, geriatrics, and geriatric psychiatry.^50^ Neighborhood-level factors were measured at the census tract level using composite index variables. Details of variable definitions and operationalization are provided in Table S4. All mediators were measured in the year of dementia onset.

### Statistical Analysis

We estimated racial and ethnic disparities in timely dementia diagnosis using multivariable logistic regression in model A, adjusted for IOM-defined covariates. Models B, C, and D sequentially added individual-level socioeconomic, neighborhood-level socioeconomic, and health care access factors as potential mediators in a stepwise manner to assess how the magnitude of disparities changed. We also estimated the unadjusted time to first diagnosis among those with delayed diagnosis and the proportion of undiagnosed across racial and ethnic groups.

To quantify the contributions of each potential mediator to the racial and ethnic disparities observed in model A, we used the general mediation approach by Imai et al.^51^ Specifically, we estimated the association between race and ethnicity and timely dementia diagnosis, and the association between race and ethnicity and each potential mediator, using logistic regression adjusted for the aforementioned covariates. We then estimated mediation effects (i.e., the indirect effect, representing the portion of the association of race and ethnicity with the outcome that operates through the mediator variable) and the proportion mediated by each factor (Methods S2). A directed acyclic graph (DAG) depicting the mediation framework is presented in Figure S1. All analyses incorporated HRS sampling weights to account for unequal probability of inclusion in the sample.

We performed several sensitivity analyses. First, to assess the robustness of our findings to the choice of mediation method, we conducted a regression-based mediation analysis using the product-of-coefficients method by Cheng et al.^52^ Second, we excluded individuals whose dementia status in HRS reverted to non-dementia after onset. Third, we restricted the sample to 2000–2016, the period for which Hudomiet et al.’s original dementia measures were fully available. Fourth, we excluded dual-eligible beneficiaries to avoid undercounting dementia diagnoses captured only in Medicaid claims.

All analyses were conducted using Stata, version 18 (StataCorp LLC) and R, version 4.3.0 (R Project for Statistical Computing). *P* < 0.05 (2-sided) was considered statistically significant. The institutional review board of Yale University reviewed and approved this study. Informed consent was waived because of minimal risk and the impracticality of obtaining consent from participants.

## RESULTS

### Sample Characteristics

The final sample included 3, 429 HRS participants (Fig. 1), representing an estimated 11,002,244 U.S. adults aged 65 or older nationwide after applying HRS sampling weights (Table S5). Just over half (53.4%) received a timely diagnosis (Table 1). Among them, the most frequently recorded codes were “Other persistent mental disorders due to conditions classified elsewhere” (23%), “Alzheimer’s disease” (22%), and “Senile dementia, uncomplicated” (15%) (Table S6).

**Table 1:** Characteristics of the Study Sample, by Timely Versus Delayed or Missed Diagnosis

**Characteristic**	**Patients, no. (%)**		
	Timely diagnosis^*^	**Delayed/missed diagnosis**^**†**^	**Full sample**
All observations, no.	1,831 (53.4)	1,598 (46.6)	3,429
Individual level			
Age, y			
65–69	87 (4.8)	170 (10.6)	257 (7.5)
70–74	137 (7.5)	153 (9.6)	290 (8.5)
75–79	292 (15.9)	256 (16.0)	548 (16.0)
80–84	423 (23.1)	362 (22.7)	785 (22.9)
≥ 85	892 (48.7)	657 (41.1)	1,549 (45.2)
Race and ethnicity			
Non-Hispanic White	1,414 (77.2)	1,123 (70.3)	2,537 (74.0)
Non-Hispanic Black	275 (15.0)	285 (17.8)	560 (16.3)
Hispanic	117 (6.4)	137 (8.6)	254 (7.4)
Non-Hispanic other	25 (1.4)	53 (3.3)	78 (2.3)
Male	590 (32.2)	626 (39.2)	1,216 (35.5)
High school educational attainment or higher	1,100 (60.1)	825 (51.6)	1,925 (56.1)
Ratio of family income to poverty threshold			
Less than 1	311 (17.0)	321 (20.1)	632 (18.4)
Between 1 and 4	1,104 (60.3)	955 (59.8)	2,059 (60.0)
Greater than or equal to 4	416 (22.7)	322 (20.2)	738 (21.5)
Number of chronic conditions^‡^, mean (SD)	2.5 (1.5)	2.4 (1.5)	2.5 (1.5)
Number of activities of daily living (ADL), mean (SD)	2.3 (2.3)	1.8 (2.0)	2.1 (2.2)
Number of instrumental activities of daily living (IADL), mean (SD)	2.7 (1.9)	1.8 (1.7)	2.3 (1.8)
Predicted latent cognition, mean (SD)	−0.6 (0.6)	−0.3 (0.4)	−0.5 (0.5)
Was seen by a dementia specialist^§^	756 (41.3)	527 (33.0)	1,283 (37.4)
Neighborhood level^¶^			
Density of physician offices/clinics			
Low density (deciles 1–2)	529 (28.9)	574 (35.9)	1,103 (32.2)
Medium density (deciles 3–7)	656 (35.8)	576 (36.0)	1,232 (35.9)
High density (deciles 8–10)	646 (35.3)	448 (28.0)	1,094 (31.9)
Density of health practitioner offices/clinics			
Low density (deciles 1–2)	596 (32.6)	678 (42.4)	1,274 (37.2)
Medium density (deciles 3–7)	589 (32.2)	488 (30.5)	1,077 (31.4)
High density (deciles 8–10)	646 (35.3)	432 (27.0)	1,078 (31.4)
Neighborhood disadvantage			
Least disadvantage (deciles 1–2)	431 (23.5)	301 (18.8)	732 (21.3)
Intermediate disadvantage (deciles 3–7)	936 (51.1)	825 (51.6)	1,761 (51.4)
Most disadvantage (deciles 8–10)	464 (25.3)	472 (29.5)	936 (27.3)
Neighborhood affluence			
Least affluent (deciles 1–2)	288 (15.7)	390 (24.4)	678 (19.8)
Intermediate affluent (deciles 3–7)	909 (49.6)	803 (50.3)	1,712 (49.9)
Most affluent (deciles 8–10)	634 (34.6)	405 (25.3)	1,039 (30.3)

^*^Timely diagnosis was defined as the presence of a clinical diagnosis of dementia, identified by an ICD-9-CM or ICD-10-CM code in Medicare claims, recorded up to 3 years prior to or 1 year following the HRS interview at which dementia was first observed

^†^Delayed/missed diagnosis was defined as the absence of a timely diagnosis, including individuals who received their first clinical dementia diagnosis more than 1 year after the HRS interview at which dementia was first observed or who never received a dementia diagnosis in Medicare claims

^‡^Details on chronic conditions are provided in Supplementary Table S3

^§^Dementia specialties included neurology, psychiatry, neuropsychiatry, geriatrics, and geriatric psychiatry

^**¶**^Details on neighborhood-level characteristics are provided in Supplementary Table S4

Of the participants, 74.0% self-identified as non-Hispanic White, 16.3% as non-Hispanic Black, and 7.4% as Hispanic (Table 2). Compared with individuals who self-identified as non-Hispanic White, individuals who were non-Hispanic Black or Hispanic were less likely to receive a timely diagnosis (55.7% vs 49.1% and 46.1%). Weighted estimates (Table S5 and Table S7) are consistent with unweighted statistics. Among those with a delayed diagnosis, the mean time from dementia onset to first recorded clinical diagnosis was 39 months overall (Figure S2), and longer for non-Hispanic Black (44 months) and Hispanic individuals (55 months) than non-Hispanic White individuals (37 months).

**Table 2:** Characteristics of the Study Sample, by Race and Ethnicity

**Characteristic**	**Patients, no. (%)**		
	**Non-Hispanic White**	**Non-Hispanic Black**	**Hispanic**
All observations, no.	2,537 (74.0)	560 (16.3)	254 (7.4)
Timely diagnosis^*^	1,414 (55.7)	275 (49.1)	117 (46.1)
Individual level			
Age, y			
65–69	138 (5.4)	74 (13.2)	35 (13.8)
70–74	196 (7.7)	57 (10.2)	25 (9.8)
75–79	372 (14.7)	112 (20.0)	52 (20.5)
80–84	601 (23.7)	115 (20.5)	50 (19.7)
≥ 85	1,230 (48.5)	202 (36.1)	92 (36.2)
Male	889 (35.0)	210 (37.5)	83 (32.7)
High school educational attainment or higher	1,663 (65.5)	190 (33.9)	37 (14.6)
Ratio of family income to poverty threshold			
Less than 1	298 (11.7)	192 (34.3)	109 (42.9)
Between 1 and 4	1,601 (63.1)	300 (53.6)	124 (48.8)
Greater than or equal to 4	638 (25.1)	68 (12.1)	21 (8.3)
Number of chronic conditions^**†**^, mean (SD)	2.4 (1.4)	2.7 (1.5)	3.0 (1.4)
Number of activities of daily living (ADL), mean (SD)	2.0 (2.2)	2.2 (2.3)	2.8 (2.3)
Number of instrumental activities of daily living (IADL), mean (SD)	2.2 (1.8)	2.3 (1.8)	2.8 (1.8)
Predicted latent cognition, mean (SD)	−0.5 (0.5)	−0.6 (0.6)	−0.5 (0.5)
Was seen by a dementia specialist^‡^	973 (38.4)	193 (34.5)	89 (35.0)
Neighborhood level^§^			
Density of physician offices/clinics			
Low density (deciles 1–2)	747 (29.4)	234 (41.8)	101 (39.8)
Medium density (deciles 3–7)	899 (35.4)	193 (34.5)	104 (40.9)
High density (deciles 8–10)	891 (35.1)	133 (23.8)	49 (19.3)
Density of health practitioner offices/clinics			
Low density (deciles 1–2)	794 (31.3)	314 (56.1)	134 (52.8)
Medium density (deciles 3–7)	822 (32.4)	146 (26.1)	83 (32.7)
High density (deciles 8–10)	921 (36.3)	100 (17.9)	37 (14.6)
Neighborhood disadvantage			
Least disadvantage (deciles 1–2)	690 (27.2)	22 (3.9)	13 (5.1)
Intermediate disadvantage (deciles 3–7)	1,462 (57.6)	187 (33.4)	80 (31.5)
Most disadvantage (deciles 8–10)	385 (15.2)	351 (62.7)	161 (63.4)
Neighborhood affluence			
Least affluent (deciles 1–2)	297 (11.7)	236 (42.1)	118 (46.5)
Intermediate affluent (deciles 3–7)	1,313 (51.8)	260 (46.4)	105 (41.3)
Most affluent (deciles 8–10)	927 (36.5)	64 (11.4)	31 (12.2)

^*^Timely diagnosis was defined as the presence of a clinical diagnosis of dementia, identified by an ICD-9-CM or ICD-10-CM code in Medicare claims, recorded up to 3 years prior to or 1 year following the HRS interview at which dementia was first observed

^†^Details on chronic conditions are provided in Supplementary Table S3

^‡^Dementia specialties included neurology, psychiatry, neuropsychiatry, geriatrics, and geriatric psychiatry

^§^Details on neighborhood-level characteristics are provided in Supplementary Table S4

### Racial and Ethnic Disparities in Timely Dementia Diagnosis

After adjusting for IOM-defined covariates, non-Hispanic Black and Hispanic individuals had significantly lower odds of receiving a timely diagnosis than non-Hispanic White individuals (OR = 0.71; 95% CI, 0.57–0.89 and OR = 0.61; 95% CI, 0.44–0.85, respectively; *P* < 0.01 for both) (Table 3). These disparities were substantially attenuated after sequentially adjusting for individual-level socioeconomic status (model B), neighborhood-level socioeconomic factors (model C), and health care access factors (model D), with odds of timely diagnosis no longer statistically significantly different across racial and ethnic groups in models B through D. In model D, adjusted ORs were 0.92 (95% CI, 0.72–1.17) for non-Hispanic Black individuals and 0.90 (95% CI, 0.64–1.26) for Hispanic individuals, compared with non-Hispanic White individuals.

**Table 3:**
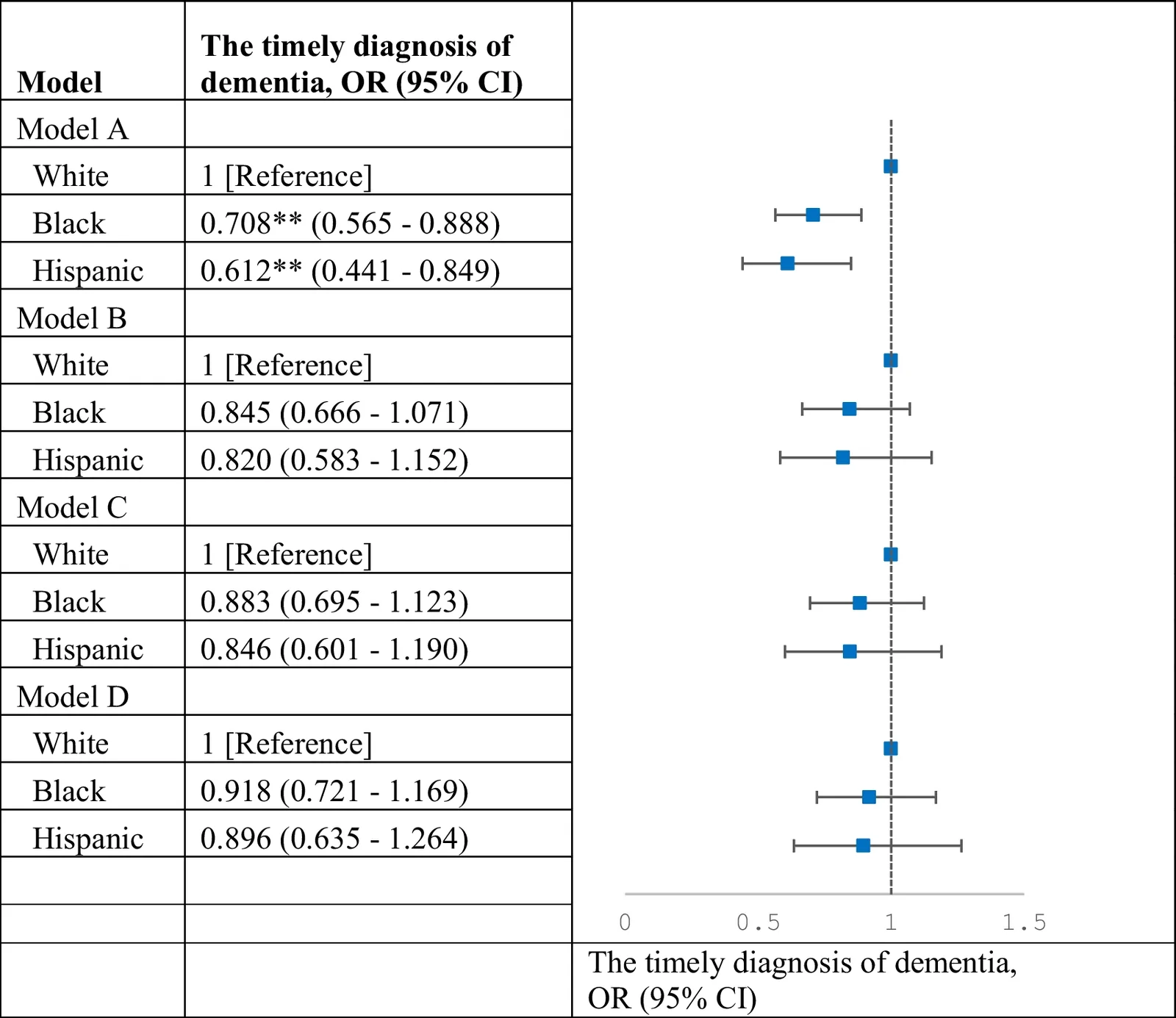
Adjusted Associations Between Race and Ethnicity and Timely Diagnosis of Dementia

**P < 0.01; *P < 0.05

Model A: adjusted for IOM-defined covariates

Model B: adjusted for IOM-defined covariates + individual-level socioeconomic status

Model C: adjusted for IOM-defined covariates + individual-level socioeconomic status + neighborhood-level socioeconomic factors

Model D: adjusted for IOM-defined covariates + individual-level socioeconomic status + neighborhood-level socioeconomic factors + health care access factors

Abbreviations: *IOM*, Institute of Medicine

Multivariable logistic regression was used to estimate racial and ethnic disparities in timely dementia diagnosis. IOM-defined covariates included non-modifiable demographics and health conditions, as described in the “Covariates” section. Individual-level socioeconomic status, neighborhood-level socioeconomic factors, and health care access factors are described in the “Potential Mediators” section

### Mediators of Timely Diagnosis

**Figure 2:**
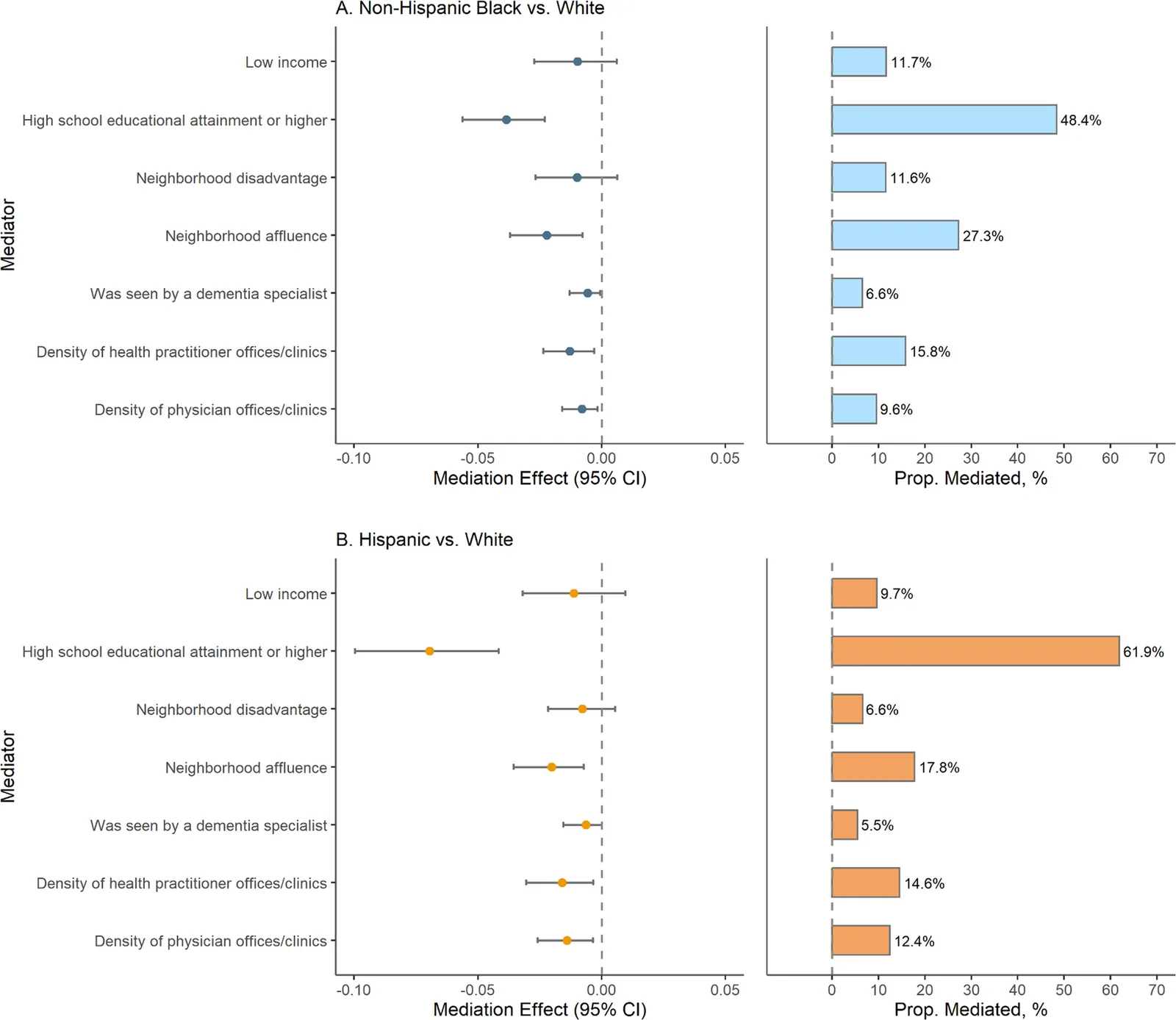
Relative effects from mediation analysis for the timely diagnosis of dementia. Notes: Panel **A** presents estimates from mediation analysis comparing non-Hispanic Black with non-Hispanic White individuals; Panel **B** presents estimates from mediation analysis comparing Hispanic with non-Hispanic White individuals. All mediator variables were operationalized as binary indicators. Low income was defined as a family income-to-poverty ratio below 1. Neighborhood disadvantage and affluence were coded as one for individuals in the least disadvantaged (disadvantage index deciles 1–2) and most affluent (affluence index deciles 8–10) neighborhoods, respectively. “Was seen by a dementia specialist” was coded as one if the individual had any office visit to a specialist prior to diagnosis. Health practitioner (not physician) and physician office/clinic density were each coded as one for individuals in neighborhoods with a density index in deciles 8–10. The *x*-axis displays indirect effects and the proportion mediated by each factor, estimated using the general mediation approach of Imai et al. (Supplementary Methods S2).

In the mediation analysis, educational attainment was the strongest mediator, explaining 48.4% (indirect effect, −0.04 [95% CI, −0.06 to −0.02]) of the disparity in timely diagnosis between non-Hispanic Black and non-Hispanic White individuals and 61.9% (indirect effect, −0.07 [95% CI, −0.10 to −0.04]) between Hispanic and non-Hispanic White individuals (Fig. 2). Neighborhood affluence was the second largest mediator, accounting for 27.3% (indirect effect, −0.02 [95% CI, −0.04 to −0.01]) and 17.8% (indirect effect, −0.02 [95% CI, −0.04 to −0.01]) of the disparities for non-Hispanic Black and Hispanic individuals, respectively. The density of health practitioner offices also explained meaningful shares of the disparities, at 15.8% (indirect effect, −0.013 [95% CI, −0.024 to −0.003]) for non-Hispanic Black individuals and 14.6% (indirect effect, −0.016 [95% CI, −0.031 to −0.003]) for Hispanic individuals, followed by the density of physician offices, at 9.6% (indirect effect, −0.008 [95% CI, −0.016 to −0.002]) for non-Hispanic Black individuals and 12.4% (indirect effect, −0.014 [95% CI, −0.026 to −0.003]) for Hispanic individuals, respectively.

Specialist evaluation also accounted for a statistically significant but modest proportion of disparities: 6.6% for non-Hispanic Black individuals (indirect effect, −0.006 [95% CI, −0.013 to −0.001]) and 5.5% for Hispanic individuals (indirect effect, −0.006 [95% CI, −0.015 to −0.000]), compared with non-Hispanic White individuals. Income and neighborhood disadvantage were not significant mediators of racial and ethnic disparities in timely dementia diagnosis.

### Sensitivity and Additional Analyses

Sensitivity analyses using the product method for mediation (Table S8), excluding individuals whose dementia status reverted after onset (Table S9; Figure S3), and restricting the sample to 2000–2016 (Table S10; Figure S4) yielded results consistent with the main findings. Excluding dual-eligible beneficiaries produced similar results, though specialist evaluation and neighborhood affluence were not significant when comparing Hispanic versus non-Hispanic White individuals (Figure S5), likely due to the reduced sample size (Table S11) and exclusion of more socioeconomically disadvantaged individuals.

## DISCUSSION

A growing body of literature has conceptualized multiple factors as potential contributors to racial and ethnic disparities in timely dementia diagnosis, including lower educational level,^18–20^ linguistic disparities,^29^ fewer socioeconomic resources,^18^ limited access to care,^26–28^ mistrust of health care providers,^21^ and physician training and concerns about misdiagnosis.^53,54^ While not all such factors could be empirically examined due to data limitations, this study is the first, to our knowledge, to leverage survey-linked claims data to quantify the contributions of individual- and neighborhood-level socioeconomic and health care access factors—commonly hypothesized drivers of disparities—within a unified mediation framework.

Consistent with existing evidence, we found that non-Hispanic Black and Hispanic individuals with dementia were less likely to receive a timely diagnosis than non-Hispanic White individuals.^8–10^ Educational attainment was the most prominent mediator of the observed disparities, while neighborhood affluence, the density of health practitioner offices, and the density of physician offices accounted for a non-trivial portion. In contrast, income and neighborhood disadvantage were not significant mediators, suggesting that differential financial resources alone do not drive diagnostic disparities.

Educational attainment of high school or above explained the largest share of disparities in timely dementia diagnosis. Non-Hispanic Black and Hispanic individuals with dementia had considerably lower proportions of high school completion than non-Hispanic White individuals. Low educational attainment is a key correlate of limited health literacy and poor dementia knowledge.^55,56^ This deficiency often impairs perception of cognitive decline, awareness of dementia and treatments,^57^ hindering individuals’ ability to recognize early symptoms and effectively navigate the health care system, ultimately reducing the likelihood of seeking timely medical evaluation. Although racial and ethnic disparities in educational attainment are deeply rooted in historical and structural inequities that cannot be addressed through resource redistribution alone,^58,59^ our findings highlight the potential relevance of dementia care interventions targeting demand-side barriers related to low educational attainment, such as dementia education programs and care navigation initiatives, to improve timely detection among minority populations.

Prior research has demonstrated that neighborhood disadvantage and rurality are associated with timely dementia diagnosis,^9^ but relies on broad contextual measures such as the Area Deprivation Index, a multidimensional evaluation of neighborhood socioeconomic conditions. Our study adds new evidence by distinguishing distinct neighborhood dimensions and examining their role in diagnosis disparities. The finding that neighborhood affluence, but not disadvantage, was a significant mediator likely reflects their distinct constructs: affluent neighborhoods are characterized by concentrations of high-income, highly educated professionals; these areas often exert greater social control and leverage over local institutions to facilitate health-promoting environments^60,61^—advantages that extend beyond the mere absence of deprivation found in typical non-disadvantaged neighborhoods. Racial and ethnic differences in exposure to affluent neighborhoods may therefore contribute to differences in dementia awareness and care-seeking behavior, underscoring the potential of place-based and community-level approaches to address disparities in timely diagnosis.

Although specialist evaluation was a significant mediator of timely diagnosis disparities, consistent with prior hypotheses regarding differential access to specialty care,^22,26^ its mediating role was notably smaller than that of non-physician health practitioner and physician office density. Moreover, fewer than 40% of patients across racial and ethnic groups had received a specialist evaluation. These findings highlight the critical role of broader clinical settings in dementia detection. Prior literature documents primary care physicians as the first point of contact for cognitive symptoms and non-physician clinicians as key contributors to early symptom recognition through sustained patient engagement.^62,63^ Given their broader geographic distribution relative to dementia specialist practices and the rapidly growing supply of non-physician primary care clinicians such as nurse practitioners,^64^ policy efforts may consider supporting these providers in underserved communities and strengthening referral pathways into specialty care.

This study had several limitations. First, we used an HRS-based prediction algorithm to determine dementia status due to the lack of clinical ascertainment of dementia onset. Any non-differential misclassification, as well as the omission of individuals identified by claims diagnoses alone, would likely attenuate estimated disparities toward the null. Second, our sample was limited to Medicare FFS beneficiaries. MA enrollees may differ systematically from FFS beneficiaries in socioeconomic status, health literacy, and care-seeking behavior, and MA plans differ from FFS in care access, delivery, and financing; our findings may therefore not generalize to MA enrollees. Third, our sample excluded other racial and ethnic groups (e.g., Asian or Pacific Islander) due to small sample sizes; findings may not generalize to these populations given potential differences in socioeconomic characteristics and health care access. Fourth, although census tract-level measures of health care access are granular, they may not fully reflect individual access circumstances or experiences within larger geographic areas. Finally, although our analysis used the statistical framework of mediation analysis, causal interpretation requires strong assumptions (described in Methods S2), including known timing of mediator measurement, that may not hold in our data. Our findings therefore identify factors that statistically account for disparities rather than causal mechanisms through which they operate.

Without a universal cognitive screening recommendation in the U.S. and given disproportionately high dementia incidence among non-Hispanic Black and Hispanic older adults, our findings point to important directions for targeted intervention to improve timely dementia detection and advance equity in dementia care.

## Supplementary Information

Below is the link to the electronic supplementary material.

Supplementary file1 (PDF 1.40 MB)

## Data Availability

Restricted Health and Retirement Study (HRS) data and HRS-Medicare linked data used in this study are available to approved researchers through the HRS Restricted Data Application and the National Institute on Aging (NIA) Data LINKAGE Program (LINKAGE), respectively, under data use agreements. National Neighborhood Data Archive (NaNDA) data are publicly available at https://nanda.isr.umich.edu/data/.

## References

[1] Alzheimer’s disease facts and figures. Vol. 19. 2023:1598–1695. Alzheimers Dement. 1552–5260. Apr. <Go to ISI>://WOS:000949266300001. https://alz-journals.onlinelibrary.wiley.com/doi/pdfdirect/10.1002/alz.13016?download=true. 10.1002/alz.13016 36918389

[2] **Hurd MD, Martorell P, Langa KM.** Monetary Costs of Dementia in the United States. New Engl J Med. 2013;369(5):489-490. 10.1056/NEJMc1305541 23902508

[3] **Stephen R, Barbera M, Peters R, et al.** Development of the first WHO guidelines for risk reduction of cognitive decline and dementia: lessons learned and future directions. Front Neurol. 2021;12:763573. 10.3389/fneur.2021.763573 PMC8577650 34764935

[4] **Giezendanner S, Monsch AU, Kressig RW, et al.** General practitioners’ attitudes towards early diagnosis of dementia: a cross-sectional survey. BMC Fam Pract. 2019;20(1):1-9. 10.1186/s12875-019-0956-1 PMC6528190 31109304

[5] Force UPST. Screening for Cognitive Impairment in Older Adults: US Preventive Services Task Force Recommendation Statement. JAMA. 2020;323(8):757-763. 10.1001/jama.2020.0435. 32096858

[6] **Amjad H, Roth DL, Sheehan OC, Lyketsos CG, Wolff JL, Samus QM.** Underdiagnosis of Dementia: an Observational Study of Patterns in Diagnosis and Awareness in US Older Adults. J Gen Intern Med. 2018;33(7):1131-1138. 10.1007/s11606-018-4377-y. PMC6025653 29508259

[7] **Chen Y, Power MC, Grodstein F, et al.** Correlates of missed or late versus timely diagnosis of dementia in healthcare settings. Alzheimers Dement. 2024;20(8):5551-5560. 10.1002/alz.14067. PMC11350028 38934297

[8] **Lin PJ, Daly AT, Olchanski N, et al.** Dementia Diagnosis Disparities by Race and Ethnicity. Med Care. 2021;59(8):679-686. 10.1097/Mlr.0000000000001577. PMC8263486 34091580

[9] **Tsoy E, Kiekhofer RE, Guterman EL, et al.** Assessment of Racial/Ethnic Disparities in Timeliness and Comprehensiveness of Dementia Diagnosis in California. Jama Neurol. 2021;78(6):657-665. 10.1001/jamaneurol.2021.0399. PMC8008426 33779684

[10] **White L, Ingraham B, Larson E, Fishman P, Park S, Coe NB.** Observational study of patient characteristics associated with a timely diagnosis of dementia and mild cognitive impairment without dementia. J Gen Intern Med. 2022;37(12):2957-2965. 10.1007/s11606-021-07169-7. PMC9485306 34647229

[11] **Davis MA, Lee KA, Harris M, et al.** Time to dementia diagnosis by race: a retrospective cohort study. J Am Geriatr Soc. 2022;70(11):3250-3259. 10.1111/jgs.18078. PMC9669160 36200557

[12] **Demirovic J, Prineas R, Loewenstein D, et al.** Prevalence of dementia in three ethnic groups: the South Florida program on aging and health. Ann Epidemiol. 2003;13(6):472-8. 10.1016/s1047-2797(02)00437-4. 12875807

[13] **Tang MX, Cross P, Andrews H, et al.** Incidence of AD in African-Americans, Caribbean Hispanics, and Caucasians in northern Manhattan. Neurology. 2001;56(1):49-56. 10.1212/wnl.56.1.49. 11148235

[14] **Potter GG, Plassman BL, Burke JR, et al.** Cognitive performance and informant reports in the diagnosis of cognitive impairment and dementia in African Americans and whites. Alzheimers Dement. 2009;5(6):445-53. 10.1016/j.jalz.2009.04.1234. PMC2805266 19896583

[15] **Fitzpatrick AL, Kuller LH, Ives DG, et al.** Incidence and prevalence of dementia in the Cardiovascular Health Study. J Am Geriatr Soc. 2004;52(2):195-204. 10.1111/j.1532-5415.2004.52058.x. 14728627

[16] **Pomara N, Imbimbo BP.** Use of Donanemab in Early Symptomatic Alzheimer Disease. Jama-J Am Med Assoc. 2023;330(23):2304-2304. 10.1001/jama.2023.21106. 38112820

[17] **Haggerty KL, Epstein-Lubow G, Stoeckle RJ, et al.** Applying an evidence-based approach to comprehensive dementia care under the new GUIDE model. Health Aff Forefront. 2023.

[18] **Mensah EA, Adjini M, Pringle-Weston B, Akosah A, Addo R.** Late dementia diagnosis among elderly minority populations in the United States: Causes, effects, and addressing inequities. Int J Transl Med Res Publ Health. 2024;8(Continuous):e010-e010.

[19] **Lim S, Mohaimin S, Min D, et al.** Alzheimer’s Disease and its Related Dementias among Asian Americans, Native Hawaiians, and Pacific Islanders: A Scoping Review. J Alzheimers Dis. 2020;77(2):523-537. 10.3233/jad-200509. PMC8638681 32675416

[20] **Connell CM, Scott Roberts J, McLaughlin SJ, Akinleye D.** Racial differences in knowledge and beliefs about Alzheimer disease. Alzheimer Dis Assoc Disord. 2009;23(2):110-6. 10.1097/WAD.0b013e318192e94d. PMC8141173 19474569

[21] **Chin AL, Negash S, Hamilton R.** Diversity and Disparity in Dementia: The Impact of Ethnoracial Differences in Alzheimer Disease. Alzheimer Dis Assoc Disord. 2011;25(3):187-195. 10.1097/WAD.0b013e318211c6c9. PMC3396146 21399486

[22] **Pérez-Palmer N, Khan A, Cheung A, Molina-Pérez X, Kozar V, Conroy ML.** Racial and Ethnic Disparities in Timeliness of Dementia Diagnosis: A Narrative Review. Curr Geriatr Rep. 2025;14(1):14.

[23] **Kim S, Yoon H, Jang Y.** Access to Primary Healthcare and Discussion of Memory Loss with a Healthcare Provider in Adults with Subjective Cognitive Decline: Does Race/Ethnicity Matter? Behav Sci. 2023;13(11):955. 10.3390/bs13110955 PMC10669110 37998701

[24] **Cox CG, Kirch M, Singer D, et al.** Older US adults’ experiences with and views about cognitive screening and blood biomarker testing for Alzheimer’s disease. Alzheimer’s & Dementia: Diagnosis, Assessment & Disease Monitoring. 2025;17(1):e70067. 10.1002/dad2.70067 PMC11736703 39822297

[25] **Lee S, Kim D, Lee H.** Examine Race/Ethnicity Disparities in Perception, Intention, and Screening of Dementia in a Community Setting: Scoping Review. Int J Environ Res Publ Health. 2022;19(14):8865. 10.3390/ijerph19148865 PMC9321249 35886711

[26] **Lewis A, Gupta A, Oh I, et al.** Association Between Socioeconomic Factors, Race, and Use of a Specialty Memory Clinic. Neurology. 2023;101(14):e1424-e1433. 10.1212/wnl.0000000000207674. PMC10573139 37532510

[27] **Murchison CF, Kennedy RE, McConathy JE, Roberson ED.** Racial differences in Alzheimer’s disease specialist encounters are associated with usage of molecular imaging and dementia medications: an enterprise-wide analysis using i2b2. J Alzheimer’s Dis. 2021;79(2):543-557. 10.3233/JAD-200796 PMC7902957 33337364

[28] **Drabo EF, Barthold D, Joyce G, Ferido P, Chui HC, Zissimopoulos J.** Longitudinal analysis of dementia diagnosis and specialty care among racially diverse Medicare beneficiaries. Alzheimer’s Dement. 2019;15(11):1402-1411. 10.1016/j.jalz.2019.07.005 PMC6874742 31494079

[29] **Silva-Rudberg JA, Carrión CI, Perez-Palmer N, et al.** Assessment of disparities in timely diagnosis and comprehensive workup of cognitive impairment between English and Spanish speakers. Am J Geriatr Psychiatry. 2024;32(7):773-786. 10.1016/j.jagp.2024.01.030 PMC11162952 38336573

[30] **Wackerbarth SB, Johnson MMS.** The Carrot and the Stick: Benefits and Barriers in Getting a Diagnosis. Alzheimer Dis Assoc Disord. 2002;16(4):213-220. 10.1097/00002093-200210000-00002 12468895

[31] **Bradford A, Kunik ME, Schulz P, Williams SP, Singh H.** Missed and delayed diagnosis of dementia in primary care: prevalence and contributing factors. Alzheimer Dis Assoc Disord. 2009;23(4):306-14. 10.1097/WAD.0b013e3181a6bebc. PMC2787842 19568149

[32] **Holt HK, Peterson CE, MacLaughlan David S, et al.** Mediation of Racial and Ethnic Inequities in the Diagnosis of Advanced-Stage Cervical Cancer by Insurance Status. Jama Netw Open. 2023;6(3):e232985-e232985. 10.1001/jamanetworkopen.2023.2985. PMC10726717 36897588

[33] **Lawson MB, Bissell MC, Miglioretti DL, et al.** Multilevel factors associated with time to biopsy after abnormal screening mammography results by race and ethnicity. Jama Oncol. 2022;8(8):1115-1126. 10.1001/jamaoncol.2022.1990 PMC9227677 35737381

[34] **Regan M, Barham T, Li Y, et al.** Risk factors underlying Racial and ethnic disparities in tuberculosis diagnosis and treatment outcomes, 2011–19: a multiple mediation analysis of National surveillance data. The Lancet Public Health. 2024;9(8):e564-e572. 10.1016/S2468-2667(24)00151-8 PMC11587887 39095133

[35] **Trinidad S, Brokamp C, Mor Huertas A, et al.** Use Of Area-Based Socioeconomic Deprivation Indices: A Scoping Review And Qualitative Analysis: Study examines socioeconomic deprivation indices. Health Affair. 2022;41(12):1804-1811. 10.1377/hlthaff.2022.00482 36469826

[36] **Spector-Bagdady K, Tang S, Jabbour S, et al.** Respecting Autonomy And Enabling Diversity: The Effect Of Eligibility And Enrollment On Research Data Demographics: Study examines the effect of eligibility and enrollment on research data demographics. Health Affair. 2021;40(12):1892-1899. 10.1377/hlthaff.2021.01197 PMC8725865 34871076

[37] **Griauzde DH, Turner CD, Othman A, et al.** A Primary Care–Based Weight Navigation Program. Jama Netw Open. 2024;7(5):e2412192-e2412192. 10.1001/jamanetworkopen.2024.12192 PMC11109771 38771575

[38] **Hudomiet P, Hurd MD, Rohwedder S.** Trends in inequalities in the prevalence of dementia in the United States. Proc Natl Acad Sci U S A. 2022;119(46):e2212205119. 10.1073/pnas.2212205119. PMC9674270 36343247

[39] **Gianattasio KZ, Ciarleglio A, Power MC.** Development of Algorithmic Dementia Ascertainment for Racial/Ethnic Disparities Research in the US Health and Retirement Study. Epidemiology. 2020;31(1):126-133. 10.1097/EDE.0000000000001101. PMC6888863 31567393

[40] Health and Retirement Study. Trends and Inequalities in the Prevalence of Dementia in the United States: Replication Package. https://hrsdata.isr.umich.edu/data-products/trends-inequalities-prevalence-dementia-us-replication-package. Accessed 7 Feb 2025.

[41] **Gianattasio KZ, Prather C, Glymour MM, Ciarleglio A, Power MC.** Racial disparities and temporal trends in dementia misdiagnosis risk in the United States. Alzheimers Dement (N Y). 2019;5:891-898. 10.1016/j.trci.2019.11.008. PMC6926355 31890853

[42] **Lee E, Gatz M, Tseng C, et al.** Evaluation of Medicare Claims Data as a Tool to Identify Dementia. J Alzheimers Dis. 2019;67(2):769-778. 10.3233/Jad-181005. PMC7164318 30689589

[43] **Davis MA, Chang CH, Simonton S, Bynum JPW.** Trends in US Medicare Decedents’ Diagnosis of Dementia From 2004 to 2017. Jama-Health Forum. 2022;3(4):e220346. 10.1001/jamahealthforum.2022.0346. PMC8976239 35977316

[44] Institute of Medicine (US) Committee on Understanding and Eliminating Racial and Ethnic Disparities in Health Care; Smedley BD﻿, Stith AY﻿, Nelson AR﻿, et al. Unequal Treatment: Confronting Racial and Ethnic Disparities in Health Care. The National Academies Press; 2003. 25032386

[45] **Cook BL, McGuire TG, Zaslavsky AM.** Measuring racial/ethnic disparities in health care: methods and practical issues. Health Serv Res. 2012;47(3 Pt 2):1232-54. 10.1111/j.1475-6773.2012.01387.x. PMC3371391 22353147

[46] **Choi SE, White J, Mertz E, Normand S-L.** Analysis of race and ethnicity, socioeconomic factors, and tooth decay among US children. Jama Netw Open. 2023;6(6):e2318425-e2318425. 10.1001/jamanetworkopen.2023.18425 PMC10273024 37318807

[47] **Fu J, Reid SA, French B, et al.** Racial disparities in COVID-19 outcomes among black and white patients with cancer. Jama Netw Open. 2022;5(3):e224304-e224304. 10.1001/jamanetworkopen.2022.4304 PMC8961318 35344045

[48] **McGuire TG, Alegria M, Cook BL, Wells KB, Zaslavsky AM.** Implementing the Institute of Medicine definition of disparities: an application to mental health care. Health Serv Res. 2006;41(5):1979-2005. 10.1111/j.1475-6773.2006.00583.x. PMC1955294 16987312

[49] **Marcondes FO, Normand S-LT, Le Cook B, et al.** Racial and Ethnic Differences in Telemedicine Use. Jama-Health Forum. 2024;5(3):e240131-e240131. 10.1001/jamahealthforum.2024.0131. PMC10960201 38517424

[50] **Drabo EF, Barthold D, Joyce G, Ferido P, Chui HC, Zissimopoulos J.** Longitudinal analysis of dementia diagnosis and specialty care among racially diverse Medicare beneficiaries. Alzheimers Dement. 2019;15(11):1402-1411. 10.1016/j.jalz.2019.07.005. PMC6874742 31494079

[51] **Imai K, Keele L, Tingley D.** A General Approach to Causal Mediation Analysis. Psychol Methods. 2010;15(4):309-334. 10.1037/a0020761. 20954780

[52] **Cheng C, Spiegelman D, Li F.** Estimating the natural indirect effect and the mediation proportion via the product method. Bmc Med Res Methodol. 20 2021;21(1):253. 10.1186/s12874-021-01425-4. PMC8606099 34800985

[53] **Withers M, Cortez‐Sanchez K, Herrera J, Ringman JM, Segal‐Gidan F.** “My backpack is so heavy”: Experiences of Latino caregivers of family with early‐onset Alzheimer’s. J Am Geriatr Soc. 2021;69(6):1539-1547. 10.1111/jgs.17091 PMC8193816 33713418

[54] **Iliffe S, Manthorpe J, Eden A.** Sooner or later? Issues in the early diagnosis of dementia in general practice: a qualitative study. Fam Pract. 2003;20(4):376-81. 10.1093/fampra/cmg407. 12876106

[55] **Cahill S, Pierce M, Werner P, Darley A, Bobersky A.** A systematic review of the public’s knowledge and understanding of Alzheimer’s disease and dementia. Alzheimer Disease Assoc Disord. 2015;29(3):255-275. 10.1097/WAD.0000000000000102 26207322

[56] **Ayalon L.** Re-examining ethnic differences in concerns, knowledge, and beliefs about Alzheimer’s disease: results from a national sample. Int J Geriatr Psychiatry. 2013;28(12):1288-95. 10.1002/gps.3959. 23559458

[57] **Zissimopoulos J, Jacobson M, Chen Y, Borson S.** Knowledge and Attitudes Concerning Aducanumab Among Older Americans After FDA Approval for Treatment of Alzheimer Disease. Jama Netw Open. 2022;5(2):e2148355-e2148355. 10.1001/jamanetworkopen.2021.48355. PMC8845005 35157058

[58] **Blanden J, Doepke M, Stuhler J.** Educational inequality. Handbook of the Economics of Education. Elsevier; 2023:405–497.

[59] **Bayer P, Charles KK.** Divergent Paths: A New Perspective on Earnings Differences Between Black and White Men Since 1940. Q J Econ. 2018;133(3):1459-1501. 10.1093/qje/qjy003.

[60] **Clarke P, Morenoff J, Debbink M, Golberstein E, Elliott MR, Lantz PM.** Cumulative Exposure to Neighborhood Context: Consequences for Health Transitions Over the Adult Life Course. Res Aging. 2014;36(1):115-142. 10.1177/0164027512470702. PMC3900407 24465068

[61] **Browning CR, Cagney KA.** Moving beyond poverty: Neighborhood structure, social processes, and health. J Health Soc Behav. 2003;44(4):552-571. 10.2307/1519799. 15038149

[62] **Warshaw GA, Bragg EJ.** Preparing the health care workforce to care for adults with Alzheimer’s disease and related dementias. Health Aff. 2014;33(4):633-641. 10.1377/hlthaff.2013.1232 24711325

[63] **Visser FCW, Kloppenburg-Lagendijk M, Hempenius L, et al.** From suspicion of cognitive decline to dementia diagnosis: a systematic review of healthcare professionals’ considerations and attitudes. Age Ageing. 2025;54(6). 10.1093/ageing/afaf176. PMC12206097 40552369

[64] **Xue Y, Smith JA, Spetz J.** Primary Care Nurse Practitioners and Physicians in Low-Income and Rural Areas, 2010-2016. JAMA. 2019;321(1):102-105. 10.1001/jama.2018.17944. PMC6583766 30620363

