# Supplementary material for: Associations of Individual- and Neighborhood-Level Factors with Racial and Ethnic Disparities in Timely Dementia Diagnosis
Source: J Gen Intern Med. 2026 May 1;41(13):3779–89. doi: 10.1007/s11606-026-10420-8 (PMC13631172; doi:10.1007/s11606-026-10420-8)
Supplement: Supplementary file 1 [file 11606_2026_10420_MOESM1_ESM.pdf]

ONLINE SUPPLEMENTARY APPENDIX

Associations of Individual- and Neighborhood-Level Factors With Racial and Ethnic Disparities in Timely Dementia Diagnosis

Figure S1: Directed Acyclic Graph for the Mediation Analysis of Racial and Ethnic Disparities in Timely Dementia Diagnosis

Figure S2: Average Delay in Dementia Diagnosis and Proportion of Undiagnosed Individuals by Race and Ethnicity

Figure S3: Relative Effects from Mediation Analysis for Timely Dementia Diagnosis, Excluding Individuals with Reverted Dementia Status

Figure S4: Relative Effects From Mediation Analysis for the Timely Diagnosis of Dementia Using the 2000-2016 Sample

Figure S5: Relative Effects From Mediation Analysis for the Timely Diagnosis of Dementia, Excluding Dual-Eligible Beneficiaries

Table S1: Individuals with Claims-Based Dementia Diagnosis Not Classified As Having Dementia by the HRS Algorithm

Table S2: ICD-9-CM and ICD-10-CM Codes Utilized for Identifying Dementia Diagnoses

Table S3: Specification of Health Condition Covariates

Table S4: Definitions and Operationalization of Potential Mediator Measures

Table S5: Weighted Sample Characteristics, by Timely versus Delayed or Missed Diagnosis

Table S6: Distribution of Dementia Diagnosis Codes Among Patients with a Timely Diagnosis

Table S7: Weighted Sample Characteristics, by Race and Ethnicity

Table S8: Mediation Analysis Using Product Methods

Table S9: Association between Race and Ethnicity and the Timely Diagnosis of Dementia, Excluding Individuals with Reverted Dementia Status

Table S10: Association between Race and Ethnicity and the Timely Diagnosis of Dementia Using the 2000-2016 Sample

Table S11: Sample Characteristics by Race and Ethnicity, Excluding Dual-Eligible Beneficiaries

Methods S1: Replication of Predicted Cognition and Dementia Measures and Application to 1998-2020

Methods S2: Additional Details on Mediation Analysis

References S1

Figure S1: Directed Acyclic Graph for the Mediation Analysis of Racial and Ethnic Disparities in Timely Dementia Diagnosis

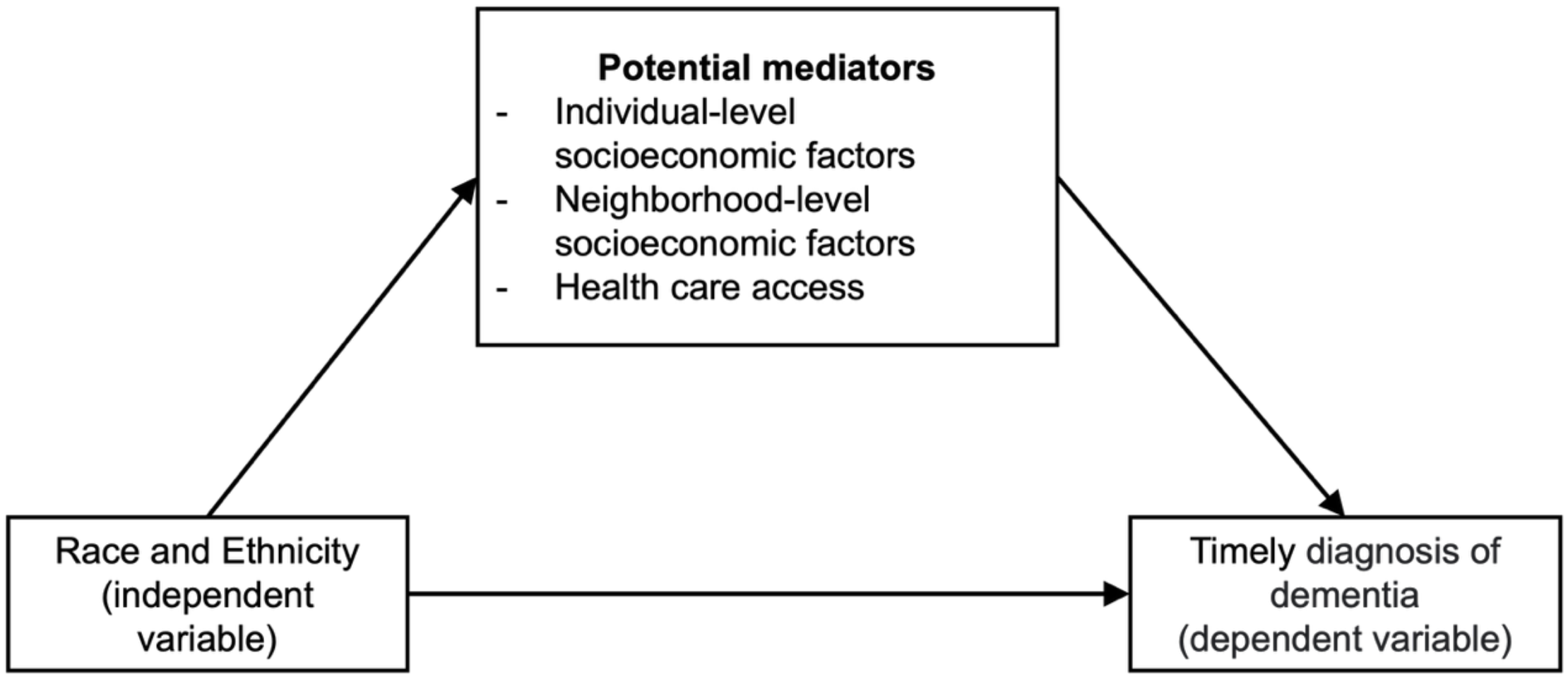

Notes: In this directed acyclic graph (DAG), race and ethnicity is the exposure and timely dementia diagnosis is the outcome. Following the Institute of Medicine (IOM) framework described in the Covariates section of the Methods, non-modifiable demographic characteristics (age, gender) and health conditions (comorbid medical conditions, ADLs/IADLs, cognition score) are treated as confounders. The mediation analysis estimated the total effect of race and ethnicity on timely dementia diagnosis, decomposing it into a direct effect and indirect effects operating through each mediator, while holding confounders constant. Each mediator was examined separately while adjusting for confounders.

Figure S2: Average Delay in Dementia Diagnosis and Proportion of Undiagnosed Individuals by Race and Ethnicity

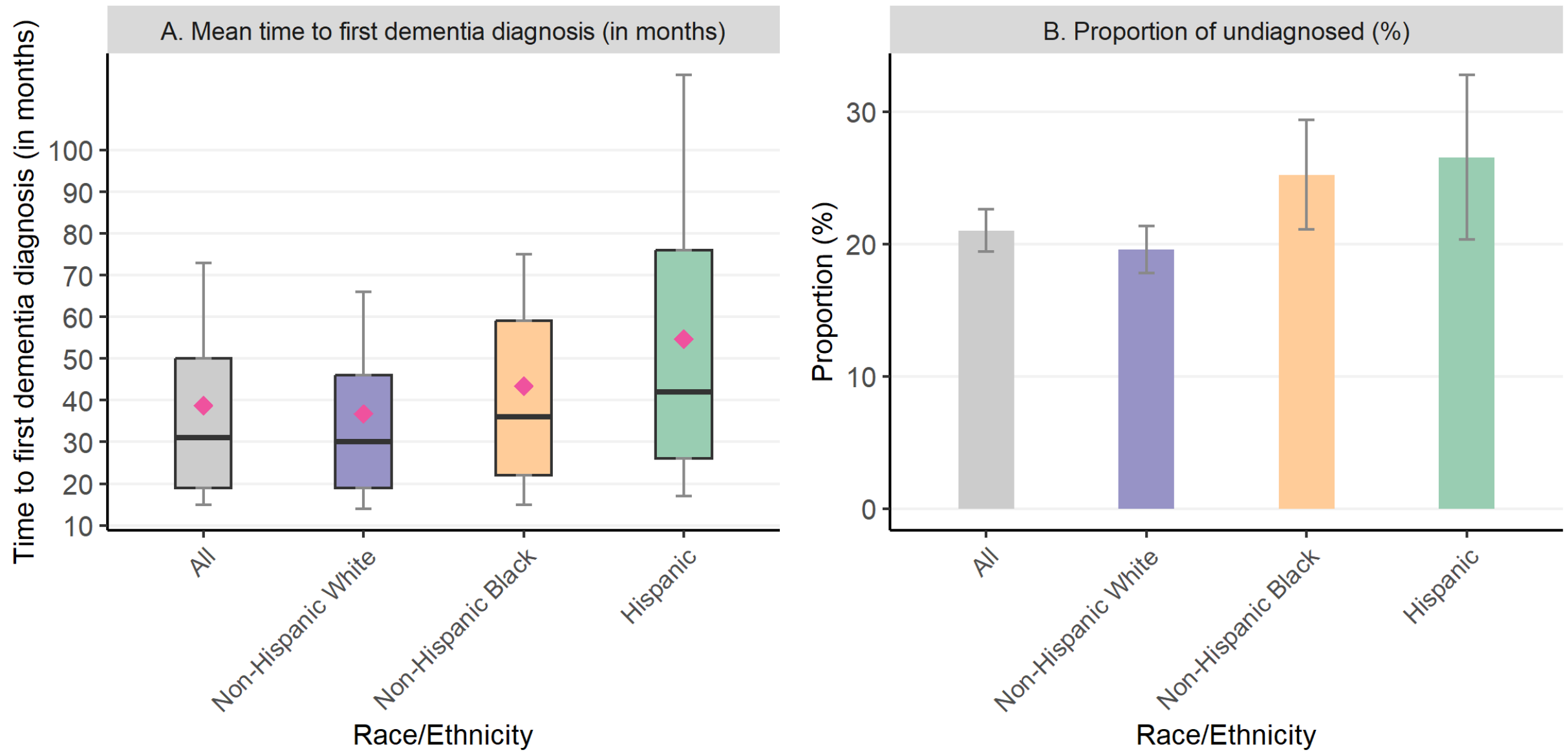

Notes: Panel A presents the unadjusted time from dementia onset (as defined by HRS) to the first recorded clinical diagnosis of dementia among individuals with a delayed diagnosis, stratified by race and ethnicity. Box boundaries indicate the 25th and 75th percentiles; whiskers, the 10th and 90th percentiles; horizontal lines, the median; and diamonds, the mean. Panel B presents the proportion of individuals with dementia who never received a diagnosis, stratified by race and ethnicity. In Panel B, whiskers represent 95% confidence intervals. All estimates were weighted using HRS analytic weights to represent U.S. adults aged 65 or older.

Figure S3: Relative Effects from Mediation Analysis for Timely Dementia Diagnosis, Excluding Individuals with Reverted Dementia Status

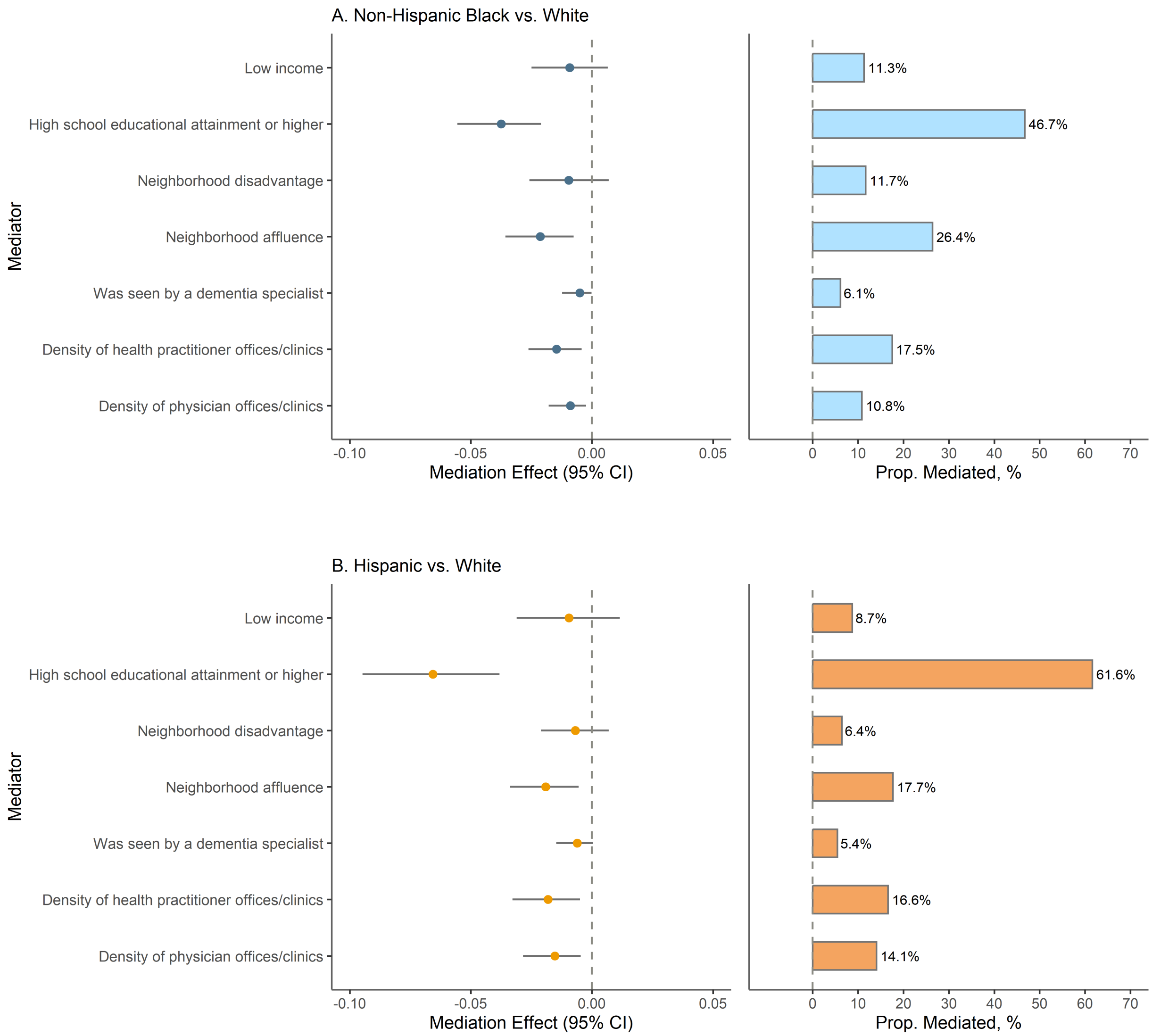

Notes: Estimates are from mediation analyses excluding individuals whose dementia status reverted to non-dementia after onset. All mediator variables were operationalized as binary indicators. Low income was defined as a family income-to-poverty ratio below 1. Neighborhood disadvantage and affluence were coded as one for individuals in the least disadvantaged (disadvantage index deciles 1-2) and most affluent (affluence index deciles 8-10) neighborhoods, respectively. "Was seen by a dementia specialist" was coded as one if the individual had any office visit to a specialist prior to diagnosis. Health practitioner (not physician) and physician office/clinic density were each coded as one for individuals in neighborhoods with a density index in deciles 8-10. The x-axis displays indirect effects and the proportion mediated by each factor, estimated using the general mediation approach of Imai et al. (Supplementary Methods S2).

Figure S4. Relative Effects From Mediation Analysis for the Timely Diagnosis of Dementia Using the 2000-2016 Sample

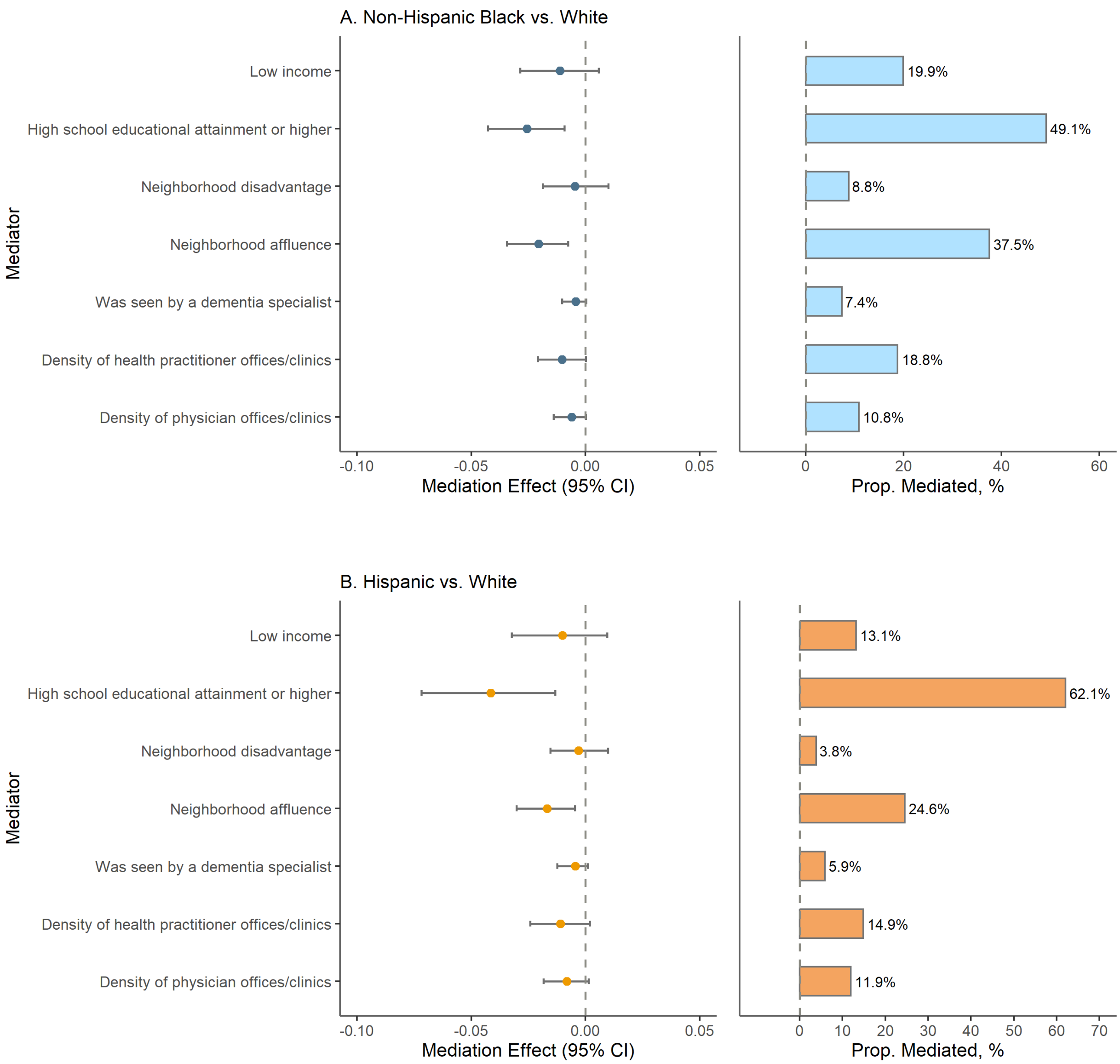

Notes: Estimates are from mediation analyses restricting the Health and Retirement Study (HRS) sample to 2000-2016. All mediator variables were operationalized as binary indicators. Low income was defined as a family income-to-poverty ratio below 1. Neighborhood disadvantage and affluence were coded as one for individuals in the least disadvantaged (disadvantage index deciles 1-2) and most affluent (affluence index deciles 8-10) neighborhoods, respectively. "Was seen by a dementia specialist" was coded as one if the individual had any office visit to a specialist prior to diagnosis. Health practitioner (not physician) and physician office/clinic density were each coded as one for individuals in neighborhoods with a density index in deciles 8-10. The x-axis displays indirect effects and the proportion mediated by each factor, estimated using the general mediation approach of Imai et al. (Supplementary Methods S2).

Figure S5: Relative Effects From Mediation Analysis for the Timely Diagnosis of Dementia, Excluding Dual-Eligible Beneficiaries

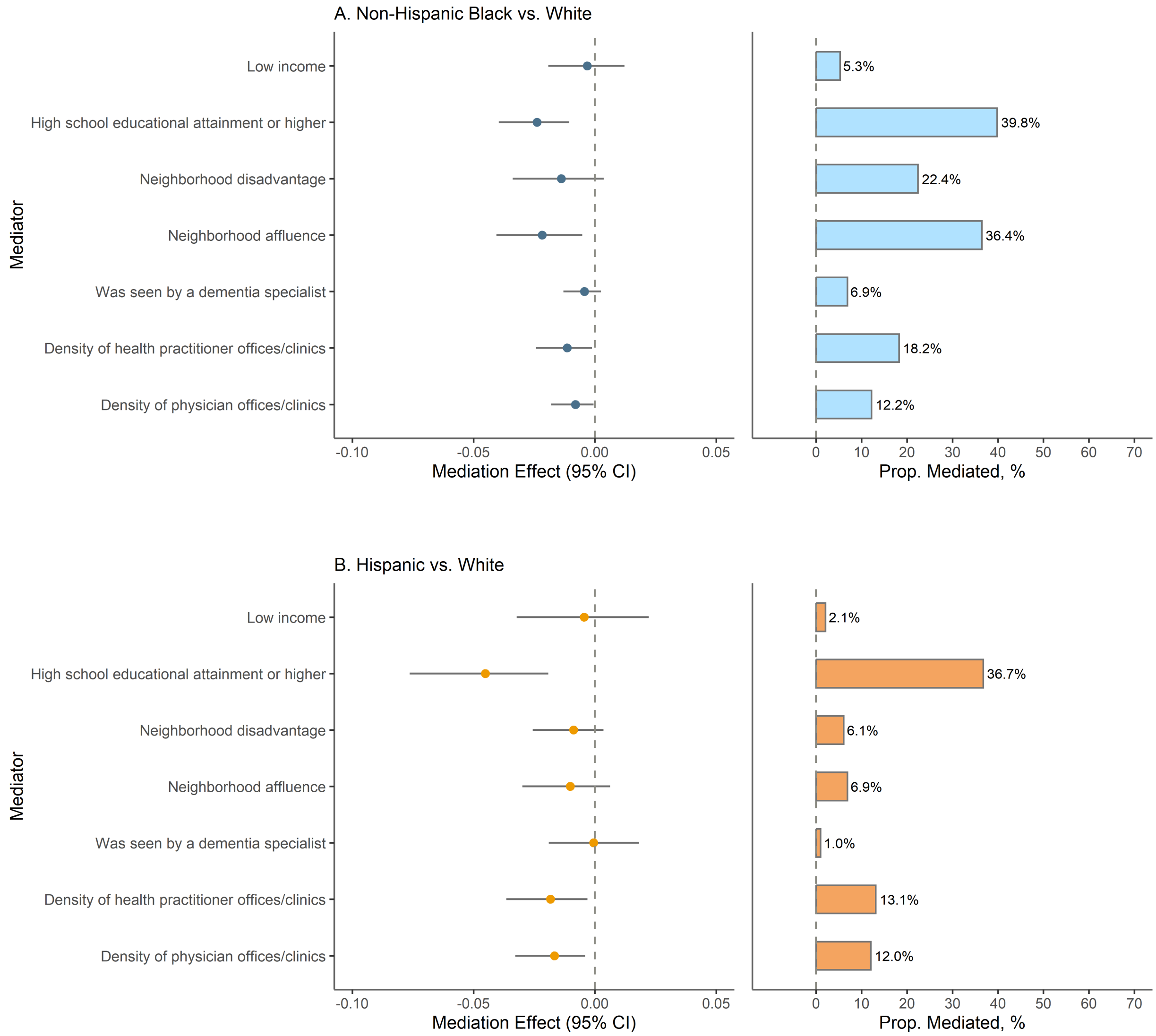

Notes: Estimates are from mediation analyses excluding dual-eligible beneficiaries. All mediator variables were operationalized as binary indicators. Low income was defined as a family income-to-poverty ratio below 1. Neighborhood disadvantage and affluence were coded as one for individuals in the least disadvantaged (disadvantage index deciles 1-2) and most affluent (affluence index deciles 8-10) neighborhoods, respectively. "Was seen by a dementia specialist" was coded as one if the individual had any office visit to a specialist prior to diagnosis. Health practitioner (not physician) and physician office/clinic density were each coded as one for individuals in neighborhoods with a density index in deciles 8-10. The x-axis displays indirect effects and the proportion mediated by each factor, estimated using the general mediation approach of Imai et al. (Supplementary Methods S2).

Table S1: Individuals with Claims-Based Dementia Diagnosis Not Classified As Having Dementia by the HRS Algorithm

**Claims-based dementia identification metrics**	**Not classified as having dementia by the HRS algorithm**		**Total person-waves**	**Total persons**
	**N (%), Person-waves**	**N (%), Persons**		
Any claims-based dementia diagnosis	3,219 (36.37)	1,613 (33.08)	8,851	4,876
≥1 inpatient, SNF, home health, or hospice claim for dementia, or ≥2 carrier/outpatient claims that are ≥7 days apart	2,007 (29.11)	985 (25.47)	6,895	3,867
Dementia claims from ≥2 distinct health care settings	1,113 (23.63)	563 (19.73)	4,710	2,854

Notes: This table is based on HRS respondents aged 65 years or older with Medicare claims linkage. It reports the number and percentage of person-waves and individuals for whom claims data indicated a dementia diagnosis but who were not classified as having dementia by the HRS algorithm (Hudomiet et al., 2022), across different claims-based identification metrics. Individuals with claims-based diagnoses who did not meet the HRS algorithmic criteria for dementia were not eligible for the analytic sample.

Table S2: ICD-9-CM and ICD-10-CM Codes Utilized for Identifying Dementia Diagnoses

Dementia			
**ICD-9-CM Code**	**Description**	**ICD-10-CM Code**	**Description**
290.0	Senile dementia, uncomplicated	F01.50	Vascular dementia without behavioral disturbance
290.10	Presenile dementia, uncomplicated	F01.51	Vascular dementia with behavioral disturbance
290.11	Presenile dementia with delirium	F02.80	Dementia in other diseases classified elsewhere without behavioral disturbance
290.12	Presenile dementia with delusional features	F02.81	Dementia in other diseases classified elsewhere with behavioral disturbance
290.13	Presenile dementia with depressive features	F03.90	Unspecified dementia without behavioral disturbance
290.20	Senile dementia with delusional features	F03.91	Unspecified dementia with behavioral disturbance
290.21	Senile dementia with depressive features	F04	Amnestic disorder due to known physiological condition
290.3	Senile dementia with delirium	F05	Delirium due to known physiological condition
290.40	Vascular dementia, uncomplicated	F06.1	Catatonic disorder due to known physiological condition
290.41	Vascular dementia with delirium	F06.8	Other specified mental disorders due to known physiological condition
290.42	Vascular dementia with delusions	G13.8	Other systemic atrophies primarily affecting the central nervous system
290.43	Vascular dementia with depressed mood	G30.0	Alzheimer's disease with early onset
294.0	Amnestic disorder in conditions classified elsewhere	G30.1	Alzheimer's disease with late onset
294.10	Dementia in conditions classified elsewhere without behavioral disturbance	G30.8	Other Alzheimer's disease
294.11	Dementia in conditions classified elsewhere with behavioral disturbance	G30.9	Alzheimer's disease, unspecified
294.20	Dementia, unspecified, without behavioral disturbance	G31.01	Pick's disease
294.21	Dementia, unspecified, with behavioral disturbance	G31.09	Other frontotemporal dementia
294.8	Other persistent mental disorders due to conditions classified elsewhere	G31.1	Senile degeneration of the brain, not elsewhere classified
331.0	Alzheimer’s disease	G31.2	Degeneration of nervous system due to alcohol
331.11	Pick’s disease	G94	Other disorders of brain in diseases classified elsewhere
331.19	Other frontotemporal dementia	R54	Senility without mention of psychosis
331.2	Senile degeneration of brain		

331.7	Cerebral degeneration in diseases classified elsewhere		
797	Senility without mention of psychosis		

Table S3: Specification of Health Condition Covariates

Variable	Definition	Source
Comorbid medical conditions	Chronic conditions were obtained from the Chronic Condition Warehouse (CCW) linked to the Health and Retirement Study (HRS) survey year at which dementia was first observed. Conditions were categorized in alignment with the HRS survey and included seven categories: hypertension, diabetes, cancer, lung disease, heart disease, stroke, and arthritis.	HRS and CCW
Latent cognition	Cognitive status was assessed using the algorithm-estimated latent cognition score developed by Hudomiet et al. (2022). Latent cognition score is a continuous measure derived from cognitive and functional assessments collected in the HRS. A detailed description of the algorithm is provided in Methods S1.	HRS
Number of ADLs	ADLs include dressing, walking across a room, bathing, eating, getting in and out of bed, and using the toilet.	HRS
Number of IADLs	IADLs include preparing meals, grocery shopping, making phone calls, taking medications, and managing money.	HRS

Notes: This table describes the health condition variables included as covariates in assessing racial and ethnic differences in timely dementia diagnosis.

Table S4: Definitions and Operationalization of Potential Mediator Measures

Measure	Definition	Source	Operationalization in mediation analysis
Income	Total household income for the last calendar year, summed across respondent and spouse earnings, pensions and annuities, SSI and Social Security Disability, Social Security retirement, unemployment and workers' compensation, other government transfers, household capital income, and other income.	Health and Retirement Study (HRS)	Binary indicator of low income, defined as a family income-to-poverty ratio below 1, derived from HRS data.
Education	Educational level, categorized as: less than high school, General Educational Development (GED), high school graduate, some college, and college or above.	Health and Retirement Study (HRS)	Binary indicator of high school graduate or higher.
Was seen by a dementia specialist	Specialist evaluation was defined as having at least one visit to a dementia specialist, including neurology, psychiatry, neuropsychiatry, geriatrics, or geriatric psychiatry, prior to diagnosis within the timely diagnosis window for those with a timely diagnosis, or within the same window for those with a delayed or missed diagnosis.	Medicare claims	A binary indicator coded as 1 if the participant had at least one visit to a dementia specialist prior to diagnosis, and 0 otherwise.
Neighborhood disadvantage	The average of four census indicators (proportion of female-headed families with children, proportion of households receiving public assistance income or food stamps, proportion of families with income below the federal poverty level, and proportion of the population aged 16 years or older who were unemployed) ranging from 0 to 1.0.	National Neighborhood Data Archive (NaNDA): Neighborhood Socioeconomic and Demographic Characteristics by Tract, United States, 2000-2020	Binary indicator coded as 1 for individuals residing in the least disadvantaged neighborhoods (disadvantage index deciles 1-2).

Neighborhood affluence	The average of three census indicators (proportion of households with income exceeding $75,000, proportion of the population aged 16 years or older employed in professional or managerial occupations, and proportion of adults with a bachelor's degree or higher) ranging from 0 to 1.0.	National Neighborhood Data Archive (NaNDA): Neighborhood Socioeconomic and Demographic Characteristics by Tract, United States, 2000-2020	Binary indicator coded as 1 for individuals residing in the most affluent neighborhoods (affluence index deciles 8-10).
Density of physician offices/clinics	The number of physician offices/clinics per 1,000 people. Physician offices and clinics were defined as establishments of licensed practitioners having the degree of M.D. (doctor of medicine) or D.O. (doctor of osteopathy) engaged in the practice of general or specialized medicine and surgery.	National Neighborhood Data Archive (NaNDA) Health Care Services by Census Tract and ZCTA, United States, 1990-2021	Binary indicator coded as 1 for individuals residing in neighborhoods with a density index in deciles 8-10.
Density of health practitioner offices/clinics	The number of health practitioner offices/clinics per 1,000 people. Health practitioner offices and clinics were defined as offices and clinics of non-physician health practitioners, including physical therapists, occupational therapists, speech pathologists, clinical psychologists, psychotherapists, registered nurses, chiropractors, podiatrists, acupuncturists, massage therapists, and other non-physician health practitioners.	National Neighborhood Data Archive (NaNDA) Health Care Services by Census Tract and ZCTA, United States, 1990-2021	Binary indicator coded as 1 for individuals residing in neighborhoods with a density index in deciles 8-10.

Notes: All measures are available during the study period (1998-2020), except for neighborhood disadvantage and affluence in 1998, which were proxied using 2000 census data given the relative stability of neighborhood-level socioeconomic characteristics over short periods.

Table S5: Weighted Sample Characteristics, by Timely versus Delayed or Missed Diagnosis

	Patients, Weighted* N (%)		
**Characteristic**	**Timely diagnosis†**	**Delayed/Missed diagnosis‡**	**Full sample**
All observations, No.	5,889,626 (53.5)	5,112,618 (46.5)	11,002,244
**Individual level**			
Age, y			
65-69	315,337 (5.4)	535,607 (10.5)	850,944 (7.7)
70-74	441,075 (7.5)	523,709 (10.2)	964,784 (8.8)
75-79	956,676 (16.2)	827,401 (16.2)	1,784,077 (16.2)
80-84	1,403,435 (23.8)	1,172,391 (22.9)	2,575,826 (23.4)
>=85	2,773,103 (47.1)	2,053,510 (40.2)	4,826,613 (43.9)
Race and Ethnicity			
Non-Hispanic White	4,946,273 (84.0)	3,977,921 (77.8)	8,924,194 (81.1)
Non-Hispanic Black	574,567 (9.8)	613,459 (12.0)	1,188,026 (10.8)
Hispanic	283,212 (4.8)	334,732 (6.5)	617,944 (5.6)
Non-Hispanic Other	85,574 (1.5)	186,506 (3.6)	272,080 (2.5)
Male	1,934,468 (32.8)	1,963,949 (38.4)	3,898,417 (35.4)
High school educational attainment or higher	3,918,252 (66.5)	2,884,668 (56.4)	6,802,920 (61.8)
Ratio of family income to poverty threshold			
Less than 1	820,234 (13.9)	907,653 (17.8)	1,727,887 (15.7)
Between 1 and 4	3,591,658 (61.0)	3,117,226 (61.0)	6,708,884 (61.0)
Greater than or equal to 4	1,477,734 (25.1)	1,087,739 (21.3)	2,565,473 (23.3)
Number of chronic conditions§, mean (SD)	2.5 (1.4)	2.4 (1.5)	2.5 (1.5)
Number of activities of daily living (ADL), mean (SD)	2.1 (2.2)	1.8 (2.0)	1.9 (2.1)
Number of instrumental activities of daily living (IADL), mean (SD)	2.5 (1.9)	1.7 (1.6)	2.1 (1.8)
Predicted latent cognition, mean (SD)	-0.5 (0.5)	-0.3 (0.3)	-0.4 (0.4)
Was seen by a dementia specialist¶	2,447,899 (41.6)	1,724,613 (33.7)	4,172,512 (37.9)
**Neighborhood level#**			
Density of physician offices/clinics			
Low density (deciles 1-2)	1,614,705 (27.4)	1,722,729 (33.7)	3,337,434 (30.3)
Medium density (deciles 3-7)	2,148,647 (36.5)	1,890,261 (37.0)	4,038,908 (36.7)
High density (deciles 8-10)	2,126,274 (36.1)	1,499,628 (29.3)	3,625,902 (33.0)
Density of health practitioner offices/clinics			
Low density (deciles 1-2)	1,779,424 (30.2)	1,946,133 (38.1)	3,725,557 (33.9)

Medium density (deciles 3-7)	1,857,986 (31.5)	1,613,549 (31.6)	3,471,535 (31.6)
High density (deciles 8-10)	2,252,216 (38.2)	1,552,936 (30.4)	3,805,152 (34.6)
Neighborhood disadvantage			
Least disadvantage (deciles 1-2)	1,476,517 (25.1)	1,076,321 (21.1)	2,552,838 (23.2)
Intermediate disadvantage (deciles 3-7)	3,145,792 (53.4)	2,794,353 (54.7)	5,940,145 (54.0)
Most disadvantage (deciles 8-10)	1,267,317 (21.5)	1,241,944 (24.3)	2,509,261 (22.8)
Neighborhood affluence			
Least affluent (deciles 1-2)	694,767 (11.8)	994,490 (19.5)	1,689,257 (15.4)
Intermediate affluent (deciles 3-7)	2,993,717 (50.8)	2,648,358 (51.8)	5,642,075 (51.3)
Most affluent (deciles 8-10)	2,201,142 (37.4)	1,469,770 (28.7)	3,670,912 (33.4)

[*] Summary statistics were weighted by HRS analytic weights to represent US adults aged 65 or older.
[†] Timely diagnosis was defined as the presence of a clinical diagnosis of dementia, identified by an ICD-9-CM or ICD-10-CM code in Medicare claims, recorded up to 3 years prior to or 1 year following the HRS interview at which dementia was first observed.
[‡] Delayed/Missed diagnosis was defined as the absence of a timely diagnosis, including individuals who received their first clinical dementia diagnosis more than 1 year after the HRS interview at which dementia was first observed or who never received a dementia diagnosis in Medicare claims.
[§] Details on chronic conditions are provided in Table S3.
[¶] Specialties classified as dementia specialties were neurology, psychiatry, neuropsychiatry, geriatrics, and geriatric psychiatry.
[#] Details on neighborhood-level characteristics are provided in Table S4.

Table S6: Distribution of Dementia Diagnosis Codes Among Patients with a Timely Diagnosis

Initial Dementia Diagnosis Codes (ICD-9-CM and ICD-10-CM)	Description	Percent of Total Frequency (%)
294.8	Other persistent mental disorders due to conditions classified elsewhere	22.99
331.0	Alzheimer’s disease	22.06
290.0	Senile dementia, uncomplicated	15.02
F03.90	Unspecified dementia without behavioral disturbance	5.35
290.20	Senile dementia with delusional features	4.70
290.40	Vascular dementia, uncomplicated	4.15
294.10	Dementia in conditions classified elsewhere without behavioral disturbance	2.84
290.10	Presenile dementia, uncomplicated	2.35
290.21	Senile dementia with depressive features	1.86
331.2	Senile degeneration of brain	1.80
G30.9	Alzheimer's disease, unspecified	1.80
797	Senility without mention of psychosis	1.64
290.20	Senile dementia with delusional features	1.42
290.3	Senile dementia with delirium	1.31
G30.1	Alzheimer's disease with late onset	1.09
294.11	Dementia in conditions classified elsewhere with behavioral disturbance	0.98
290.43	Vascular dementia with depressed mood	0.87
F03.91	Unspecified dementia with behavioral disturbance	0.87
R54	Senility without mention of psychosis	0.82
290.41	Vascular dementia with delirium	0.76
F01.50	Vascular dementia without behavioral disturbance	0.71
F02.80	Dementia in other diseases classified elsewhere without behavioral disturbance	0.71
294.21	Dementia, unspecified, with behavioral disturbance	0.55
F05	Delirium due to known physiological condition	0.44
290.42	Vascular dementia with delusions	0.38
294.0	Amnestic disorder in conditions classified elsewhere	0.38

G31.1	Senile degeneration of the brain, not elsewhere classified	0.38
290.11	Presenile dementia with delirium	0.27
F01.51	Vascular dementia with behavioral disturbance	0.27
F02.81	Dementia in other diseases classified elsewhere with behavioral disturbance	0.27
290.13	Presenile dementia with depressive features	0.22
290.12	Presenile dementia with delusional features	0.16
G30.8	Other Alzheimer's disease	0.16
331.7	Cerebral degeneration in diseases classified elsewhere	0.11
331.11	Pick’s disease	0.05
F04	Amnestic disorder due to known physiological condition	0.05
G30.0	Alzheimer's disease with early onset	0.05
G31.09	Other frontotemporal dementia	0.05
G94	Other disorders of brain in diseases classified elsewhere	0.05

Notes: For each individual, the most recent Medicare claim containing a dementia diagnosis code within the timely diagnosis window (3 years pre- to 1 year post-dementia onset as defined in the HRS) was used for this frequency table. When multiple dementia codes were present within that claim, the first listed code was used.

Table S7: Weighted Sample Characteristics, by Race and Ethnicity

Characteristic	Patients, Weighted N* (%) Non-Hispanic White	Non-Hispanic Black	Hispanic
All observations, No.	8,924,194 (81.1)	1,188,026 (10.8)	617,944 (5.6)
Timely diagnosis†	4,946,273 (55.4)	574,567 (48.4)	283,212 (45.8)
**Individual level**			
Age, y			
65-69	551,013 (6.2)	179,411 (15.1)	94,083 (15.2)
70-74	733,216 (8.2)	141,702 (11.9)	55,289 (8.9)
75-79	1,380,128 (15.5)	236,495 (19.9)	124,647 (20.2)
80-84	2,155,506 (24.2)	216,584 (18.2)	121,017 (19.6)
>=85	4,104,331 (46.0)	413,834 (34.8)	222,908 (36.1)
Male	3,108,485 (34.8)	471,978 (39.7)	196,465 (31.8)
High school educational attainment or higher	6,151,746 (68.9)	437,541 (36.8)	82,175 (13.3)
Ratio of family income to poverty threshold			
Less than 1	983,208 (11.0)	396,206 (33.3)	242,443 (39.2)
Between 1 and 4	5,636,233 (63.2)	638,062 (53.7)	309,101 (50.0)
Greater than or equal to 4	2,304,753 (25.8)	153,758 (12.9)	66,400 (10.7)
Number of Chronic Conditions‡, mean (SD)	2.386 (1.440)	2.620 (1.514)	2.994 (1.346)
Number of activities of daily living (ADL)	1.879 (2.121)	1.999 (2.194)	2.724 (2.296)
Number of instrumental activities of daily living (IADL)	2.092 (1.801)	2.135 (1.806)	2.777 (1.779)
Predicted latent cognition	-0.380 (0.401)	-0.463 (0.486)	-0.421 (0.510)
Was seen by a dementia specialist¶	3,449,155 (38.6)	408,116 (34.4)	216,790 (35.1)
**Neighborhood level#**			
Density of physician offices/clinics			
Low density (deciles 1-2)	2,533,361 (28.4)	484,189 (40.8)	252,492 (40.9)
Medium density (deciles 3-7)	3,243,707 (36.3)	407,905 (34.3)	258,001 (41.8)
High density (deciles 8-10)	3,147,126 (35.3)	295,932 (24.9)	107,451 (17.4)
Density of health practitioner offices/clinics			
Low density (deciles 1-2)	2,656,162 (29.8)	659,322 (55.5)	302,599 (49.0)
Medium density (deciles 3-7)	2,855,065 (32.0)	303,207 (25.5)	224,157 (36.3)
High density (deciles 8-10)	3,412,967 (38.2)	225,497 (19.0)	91,188 (14.8)
Neighborhood disadvantage			
Least disadvantage (deciles 1-2)	2,450,089 (27.5)	41,441 (3.5)	33,493 (5.4)

Intermediate disadvantage (deciles 3-7)	5,219,889 (58.5)	391,710 (33.0)	214,317 (34.7)
Most disadvantage (deciles 8-10)	1,254,216 (14.1)	754,875 (63.5)	370,134 (59.9)
Neighborhood affluence			
Least affluent (deciles 1-2)	879,317 (9.9)	462,753 (39.0)	267,468 (43.3)
Intermediate affluent (deciles 3-7)	4,661,561 (52.2)	581,563 (49.0)	268,086 (43.4)
Most affluent (deciles 8-10)	3,383,316 (37.9)	143,710 (12.1)	82,390 (13.3)

[*] Summary statistics were weighted by HRS analytic weights to represent US adults aged 65 or older.
[†] Timely diagnosis was defined as the presence of a clinical diagnosis of dementia, identified by an ICD-9-CM or ICD-10-CM code in Medicare claims, recorded up to 3 years prior to or 1 year following the HRS interview at which dementia was first observed.
[‡] Details on chronic conditions are provided in Table S3.
[¶] Specialties classified as dementia specialties were neurology, psychiatry, neuropsychiatry, geriatrics, and geriatric psychiatry.
[#] Details on neighborhood-level characteristics are provided in Table S4.

Table S8: Mediation Analysis Using Product Methods

	Black vs White		Hispanic vs White	
**Mediators**	**Natural indirect effect, (95% CI)**	**Mediation proportion (%)**	**Natural indirect effect, (95% CI)**	**Mediation proportion (%)**
Low income	-0.03 (-0.08, 0.02)	9.60	-0.04 (-0.11, 0.03)	8.00
High school educational attainment or higher	-0.15 (-0.2, -0.1)	43.19	-0.25 (-0.34, -0.16)	49.34
Neighborhood disadvantage	-0.05 (-0.1, -0.01)	14.57	-0.04 (-0.09, 0)	8.44
Neighborhood affluence	-0.12 (-0.17, -0.08)	34.66	-0.1 (-0.16, -0.05)	20.77
Was seen by a dementia specialist	-0.01 (-0.04, 0)	4.54	-0.01 (-0.03, 0.01)	2.43
Density of health practitioner offices/clinics	-0.07 (-0.11, -0.04)	19.77	-0.09 (-0.13, -0.05)	17.43
Density of physician offices/clinics	-0.04 (-0.07, -0.02)	11.72	-0.06 (-0.1, -0.03)	12.46

Notes: The table presents the mediation analysis results using the *Product Method* by Cheng, Spiegelman, and Li[1], based on our main analysis sample. All mediator variables were operationalized as binary indicators. Low income was defined as a family income-to-poverty ratio below 1. Neighborhood disadvantage and affluence were coded as one for individuals in the least disadvantaged (disadvantage index deciles 1-2) and most affluent (affluence index deciles 8-10) neighborhoods, respectively. "Was seen by a dementia specialist" was coded as one if the individual had any office visit to a specialist prior to diagnosis. Health practitioner (not physician) and physician office/clinic density were each coded as one for individuals in neighborhoods with a density index in deciles 8-10. The x-axis displays indirect effects and the proportion mediated by each factor, estimated using the general mediation approach of Imai et al. (Supplementary Methods S2).

Table S9: Association between Race and Ethnicity and the Timely Diagnosis of Dementia, Excluding Individuals with Reverted Dementia Status

Panel A: Proportion of individuals receiving timely diagnosis of dementia*

Race and Ethnicity	Unadjusted, N (%)	Adjusted, Weighted N (%)
Total sample	3,370	10,812,856
White	1,405 (56.2)	4,912,097 (55.9)
Black	271 (49.5)	568,424 (49.0)
Hispanic	115 (47.5)	279,134 (47.4)

Panel B: Adjusted association of race and ethnicity with the timely diagnosis of dementia†

Adjusted Model	OR (95% CI)
Model A	
White	1 [Reference]
Black	0.707** (0.563–0.889)
Hispanic	0.628** (0.449–0.879)
Model B	
White	1 [Reference]
Black	0.837 (0.658–1.064)
Hispanic	0.825 (0.583–1.168)
Model C	
White	1 [Reference]
Black	0.874 (0.685–1.115)
Hispanic	0.850 (0.600–1.205)
Model D	
White	1 [Reference]
Black	0.912 (0.714–1.165)
Hispanic	0.907 (0.638–1.289)

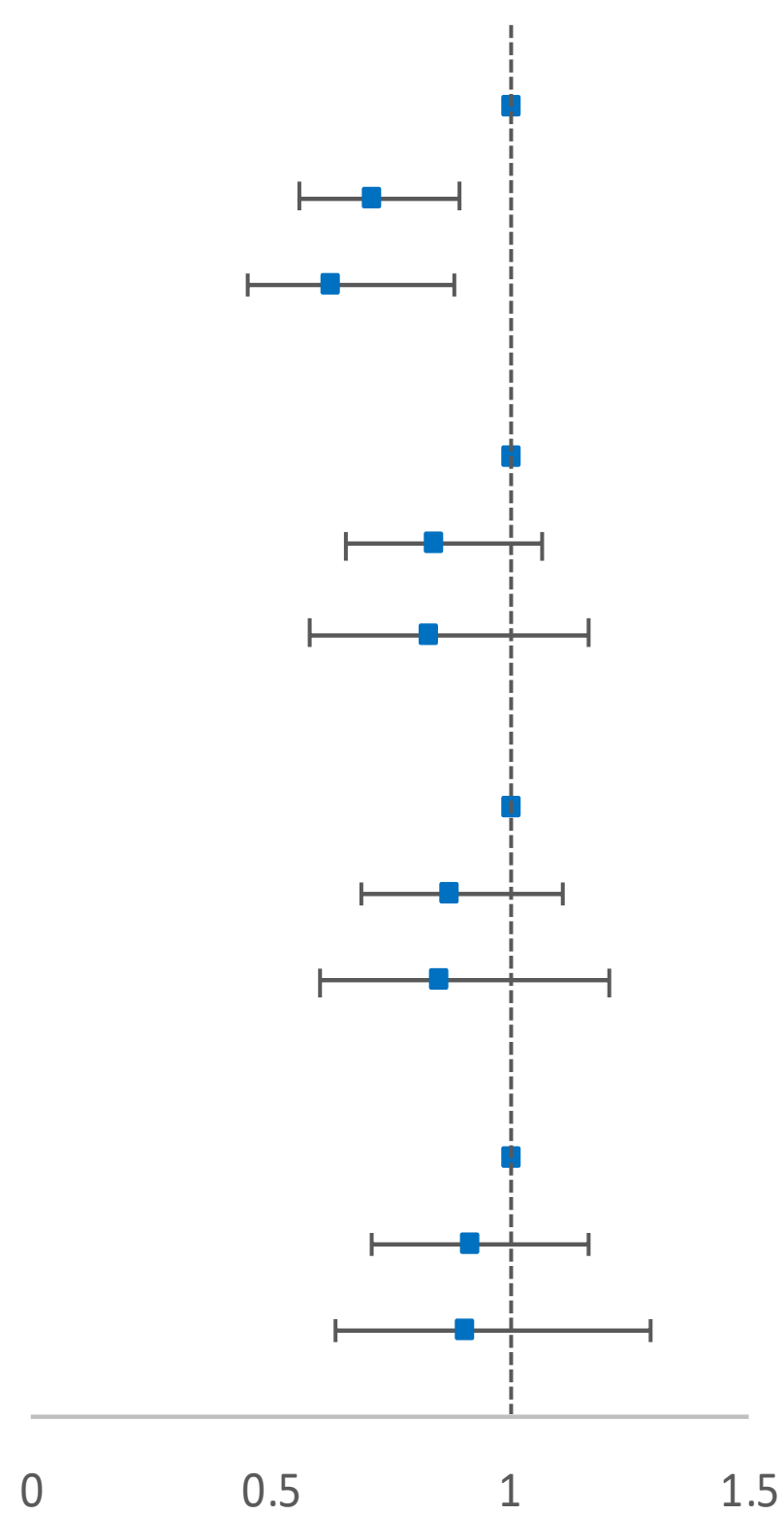

The timely diagnosis of dementia, OR (95% CI)

** p<0.01, * p<0.05, † 0<0.1

Model A: Adjusted for IOM-defined covariates
Model B: Adjusted for IOM-defined covariates + individual-level socioeconomic status
Model C: Adjusted for IOM-defined covariates + individual-level socioeconomic status + neighborhood-level socioeconomic factors
Model D: Adjusted for IOM-defined covariates + individual-level socioeconomic status + neighborhood-level socioeconomic factors + health care access factors

Notes: Estimates are from sensitivity analyses excluding individuals whose dementia status reverted to non-dementia after onset. Weighted numbers reflect the estimated number of U.S. older adults represented by the HRS participants after applying survey weights.

[*] Panel A presents both unadjusted and adjusted proportions of individuals receiving a timely dementia diagnosis in the overall population and by race and ethnicity. Adjusted proportions were estimated using predictive margins from Model A.

[†] Panel B presents the adjusted association between race and ethnicity and timely dementia diagnosis from multivariable logistic regression models, sequentially adjusting for additional variables from model A through model D. IOM-defined covariates included unmodifiable demographics and health conditions, as described in the *Covariates* section. Individual-level socioeconomic status, neighborhood-level socioeconomic factors, and health care access factors are described in the *Potential Mediators* section.

Table S10: Association between Race and Ethnicity and the Timely Diagnosis of Dementia Using the 2000-2016 Sample

Panel A: Proportion of individuals receiving timely diagnosis of dementia[*]

Race and Ethnicity	Unadjusted, N (%)	Adjusted, Weighted N (%)
Total sample	2,989	10,123,382
White	1,251 (56.8)	4,660,687 (57.1)
Black	257 (53.2)	584,427 (51.7)
Hispanic	117 (50.4)	296,139 (50.6)

Panel B: Adjusted association of race and ethnicity with the timely diagnosis of dementia[†]

Adjusted Model	OR (95% CI)
Model A	
White	1 [Reference]
Black	0.785* (0.619 - 0.996)
Hispanic	0.747† (0.532 - 1.049)
Model B	
White	1 [Reference]
Black	0.890 (0.694 - 1.142)
Hispanic	0.904 (0.635 - 1.286)
Model C	
White	1 [Reference]
Black	0.923 (0.717 - 1.188)
Hispanic	0.923 (0.647 - 1.317)
Model D	
White	1 [Reference]
Black	0.956 (0.742 - 1.231)
Hispanic	0.962 (0.675 - 1.371)

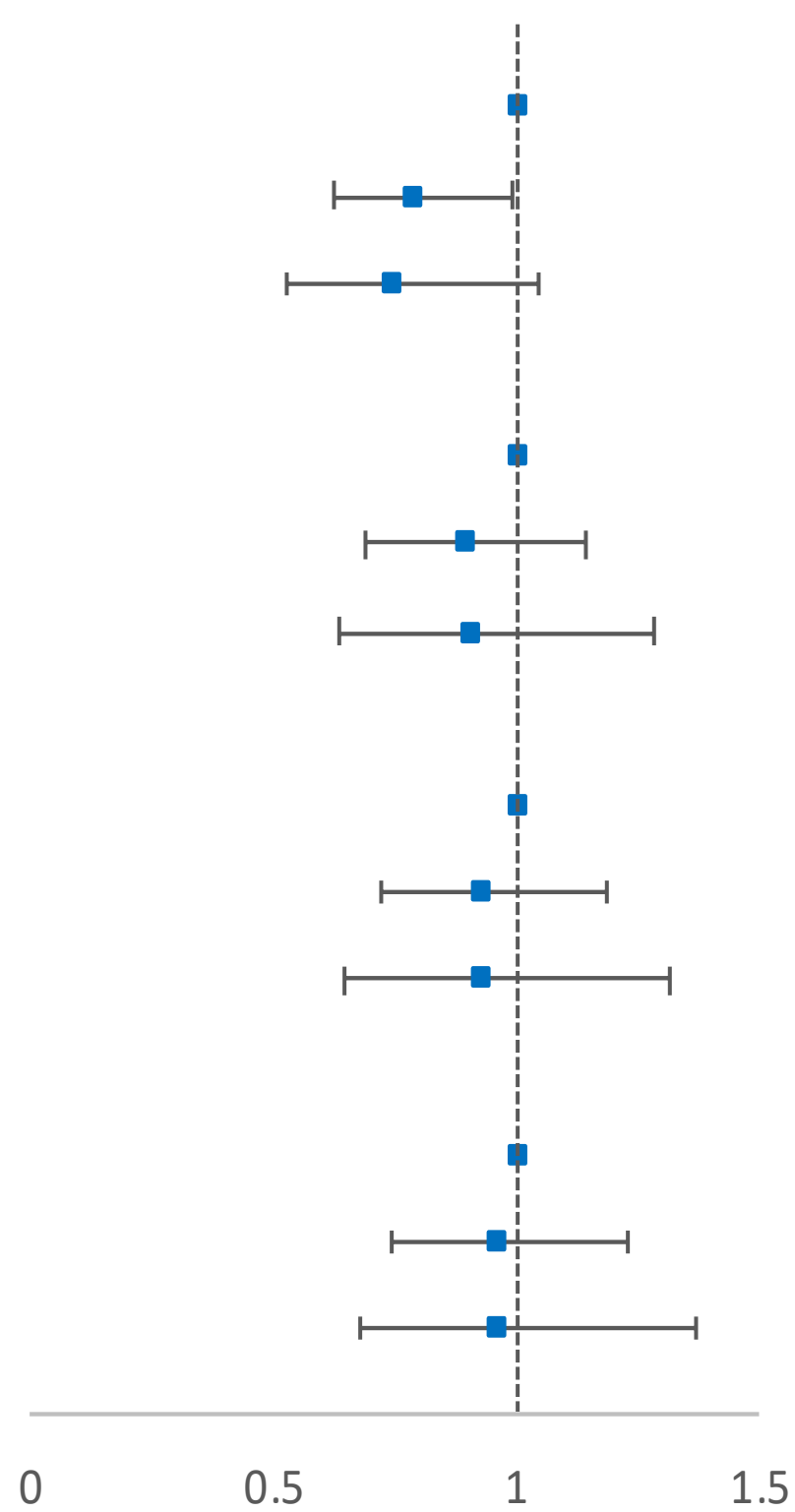

The timely diagnosis of dementia, OR (95% CI)

** p<0.01, * p<0.05, † 0<0.1

Model A: Adjusted for IOM-defined covariates
Model B: Adjusted for IOM-defined covariates + individual-level socioeconomic status
Model C: Adjusted for IOM-defined covariates + individual-level socioeconomic status + neighborhood-level socioeconomic factors
Model D: Adjusted for IOM-defined covariates + individual-level socioeconomic status + neighborhood-level socioeconomic factors + health care access factors

Notes: Estimates are from sensitivity analyses restricting the Health and Retirement Study (HRS) sample to 2000-2016. Weighted numbers reflect the estimated number of U.S. older adults represented by the HRS participants after applying survey weights.

[*] Panel A presents both unadjusted and adjusted proportions of individuals receiving a timely dementia diagnosis in the overall population and by race and ethnicity. Adjusted proportions were estimated using predictive margins from Model A.

[†] Panel B presents the adjusted association between race and ethnicity and timely dementia diagnosis from multivariable logistic regression models, sequentially adjusting for additional variables from model A through model D. IOM-defined covariates included unmodifiable demographics and health conditions, as described in the *Covariates* section. Individual-level socioeconomic status, neighborhood-level socioeconomic factors, and health care access factors are described in the *Potential Mediators* section.

Table S11: Sample Characteristics by Race and Ethnicity, Excluding Dual-Eligible Beneficiaries

	Patients, No (%)		
Characteristic	Non-Hispanic White	Non-Hispanic Black	Hispanic
All observations, No.	2,032 (84.9)	268 (11.2)	56 (2.3)
Timely diagnosis*	1,102 (54.2)	133 (49.6)	24 (42.9)
**Individual level**			
Age, mean (SD)	83.9 (7.5)	80.8 (8.1)	78.3 (9.3)
Male	766 (37.7)	121 (45.1)	27 (48.2)
High school educational attainment or higher	1,426 (70.2)	133 (49.6)	21 (37.5)
Ratio of family income to poverty threshold			
Less than 1	144 (7.1)	51 (19.0)	17 (30.4)
Between 1 and 4	1,284 (63.2)	164 (61.2)	28 (50.0)
Greater than or equal to 4	604 (29.7)	53 (19.8)	11 (19.6)
Number of Chronic Conditions†, mean (SD)	2.274 (1.414)	2.489 (1.505)	2.482 (1.501)
Number of activities of daily living (ADL), mean (SD)	1.782 (2.079)	1.735 (2.092)	1.804 (2.178)
Number of instrumental activities of daily living (IADL), mean (SD)	2.077 (1.831)	2.037 (1.832)	2.018 (1.773)
Predicted latent cognition, mean (SD)	-0.447 (0.508)	-0.499 (0.539)	-0.412 (0.583)
Was seen by a dementia specialist‡	767 (37.7)	99 (36.9)	22 (39.3)
**Neighborhood level§**			
Density of physician offices/clinics			
Low density (deciles 1-2)	591 (29.1)	112 (41.8)	22 (39.3)
Density of health practitioner offices/clinics			
Low density (deciles 1-2)	608 (29.9)	163 (60.8)	31 (55.4)
Neighborhood disadvantage			
Most disadvantage (deciles 8-10)	273 (13.4)	179 (66.8)	21 (37.5)
Neighborhood affluence			
Least affluent (deciles 1-2)	212 (10.4)	117 (43.7)	15 (26.8)

* Timely diagnosis was defined as the presence of a clinical diagnosis of dementia, identified by an ICD-9-CM or ICD-10-CM code in Medicare claims, recorded up to 3 years prior to or 1 year following the HRS interview at which dementia was first observed.
† Details on chronic conditions are provided in Supplementary Table S3.
‡ Dementia specialties included neurology, psychiatry, neuropsychiatry, geriatrics, and geriatric psychiatry.
§ To comply with Medicare data use agreements regarding minimum cell sizes, certain age and neighborhood-level subcategories could not be reported separately in this restricted sample.

Methods S1: Replication of Predicted Cognition and Dementia Measures and Application to 1998-2020

We replicated the methodology developed by Hudomiet et al. for creating predicted cognition and dementia measures, closely following their statistical approach and implementing their replication package[2]. This allowed us to extend these measures to 1998-2020, as the original study only covered 2000-2016.

The algorithm incorporated participants' performance on seven aggregated HRS cognitive measures into its prediction model for cognition. These measures included subjective limitations (the sum of self-rated memory, instrumental activities of daily living [IADLs], and self-rated health), immediate and delayed word recall, serial 7 subtraction, a mental status composite (backward counting, date naming, naming the president, cactus, and scissors), interviewer-rated cognitive limitations, and proxy-reported cognitive problems (e.g., getting lost, wandering, inability to be left alone, or seeing or hearing things).

These composite measures entered a longitudinal latent-variable model in which observed cognitive test performance is modeled as a function of an unobserved, person-wave–specific latent cognition that evolves with observed covariates and person-specific random effects. The clinical diagnosis of dementia in the Aging, Demographics and Memory Study (ADAMS) sample is used to calibrate the HRS cognitive tests to measure dementia. In ADAMS, a subsample of HRS respondents underwent a 3- to 4-hour in-home clinical assessment for dementia, and the final diagnosis was determined by an expert panel. The model was estimated using the Markov Chain Monte Carlo method using 2,000,000 simulation draws, and the first 10% burn-in draws were discarded.

After estimation, dementia classification for each person-wave observation was determined based on the latent cognition scale. Individuals were classified as having dementia if latent cognition fell below 0, cognitively impaired but not demented (CIND) if latent cognition was between 0 and 1, and cognitively normal if latent cognition exceeded 1. These cutoff values are normalizations within the latent cognition scale. The data and methodological details are described in their original paper, *Trends in inequalities in the prevalence of dementia in the United States.* (Hudomiet et al., 2022)[3].

All variables required for replication were available from 1998 to 2020, except for interviewers' assessments of cognitive limitations in proxy interviews, which were not collected in 1998. To address this, we used the 2000 assessment as a proxy measure for 1998. In addition, beginning in 2018, the HRS introduced web-based cognitive interviews, with 2016 respondents randomly assigned to phone or web-based modes. Some cognitive measures collected through web interviews differed in implementation, measurement, or elicitation. This change in data collection methods may have introduced bias in dementia prevalence estimates for 2018 and 2020. As a sensitivity analysis, we restricted the sample to 2000-2016, when the original measures were fully available, and repeated our primary analysis.

Regardless of the potential interview mode effects described above, the age-adjusted dementia prevalence shows a consistent declining trend between 1998 and 2020, a pattern consistent with

prior studies documenting declining dementia incidence and prevalence[4-6]. Birth cohort effects may play a role, as earlier cohorts experienced major social and economic disruptions that may have increased dementia risk[4]. Reductions in smoking, improved access to education, advances in nutrition, and better management of cardiovascular risk factors have been associated with lower dementia prevalence in more recent cohorts[5-8].

Below, we present our re-created dementia prevalence estimates in comparison with the originally released estimates for the comparable years 2000–2016. Overall, our re-created estimates closely tracked the original estimates.

Age-adjusted prevalence of dementia from 1998 to 2020 for total population, Age 65+

	Estimates by Hudomiet et al.		Re-created Estimates	
	Mean	95% CI	Mean	95% CI
1998	NA	NA	0.100	0.1 [0.094, 0.105]
2000	0.122	[0.117, 0.127]	0.117	0.117 [0.112, 0.122]
2002	0.109	[0.104, 0.114]	0.106	0.106 [0.101, 0.111]
2004	0.102	[0.097, 0.106]	0.101	0.101 [0.096, 0.106]
2006	0.098	[0.093, 0.103]	0.098	0.098 [0.093, 0.103]
2008	0.095	[0.09, 0.1]	0.094	0.094 [0.09, 0.099]
2010	0.093	[0.087, 0.098]	0.091	0.091 [0.086, 0.096]
2012	0.091	[0.086, 0.096]	0.089	0.089 [0.083, 0.094]
2014	0.087	[0.082, 0.093]	0.086	0.086 [0.08, 0.091]
2016	0.085	[0.079, 0.091]	0.084	0.084 [0.078, 0.089]
2018	NA	NA	0.077	0.077 [0.072, 0.082]
2020	NA	NA	0.070	0.07 [0.065, 0.075]

* Sample: Health and Retirement Study, Age 65+. The estimates by Hudomiet et al. (2000–2016) included 97,629 person-year observations; the replicated estimates (1998-2020) included 126,428 person-year observations.

Methods S2: Additional Details on Mediation Analysis

To quantify the relative contributions of individual- and neighborhood-level factors in explaining the racial and ethnic disparities observed in Model A, we constructed a series of mediation models. The mediation was conducted using a model-based approach proposed by Imai et al[9].

First, we fit a mediator model in which each potential mediator was regressed on race and ethnicity and covariates, including unmodifiable demographics and health conditions. Each mediator was modeled separately. We then fit an outcome model for receipt of a timely dementia diagnosis, with explanatory variables including the mediator, race and ethnicity, and the same covariates as those used in the mediator model. Logistic regression was used for both the mediator and outcome models. Race and ethnicity was modeled as two separate binary indicators: one comparing non-Hispanic White with non-Hispanic Black individuals, and the other comparing non-Hispanic White with Hispanic individuals, each analyzed in a separate mediation model.

When comparing non-Hispanic Black and non-Hispanic White individuals, we first generated two sets of predicted mediator values for each observation from the mediator model: one under the condition of being non-Hispanic Black and one under the condition of being non-Hispanic White. In the outcome model, we then generated two sets of outcome predictions for each observation from fixing race and ethnicity: first using the mediator value predicted under the non-Hispanic Black condition, and second using the mediator value predicted under the non-Hispanic White condition. The indirect effect was calculated as the average difference across observations between these two outcome predictions. The same approach was applied when comparing Hispanic and non-Hispanic White individuals.

Confidence intervals were calculated using heteroskedasticity-consistent standard errors. The proportion mediated by each mediator was then computed as the percentage of the total effect (i.e., the overall association between race and ethnicity and timely dementia diagnosis, adjusted for the covariates) explained by the corresponding indirect effect. All mediation analyses were implemented in R using the mediation package[10].

The identification assumption for the causal mediation analysis is sequential ignorability, which requires (1) that exposure is statistically independent of potential outcomes and potential mediators, conditional on a set of baseline covariates gathered before the exposure; and (2) that there are no unmeasured variables that confound the relationship between the mediator and the outcome. These are strong assumptions and might not be strictly met in the observational study, where exposure is not randomized, and unobserved mediator-outcome confounders may exist. Thus, we do not pursue a strong causal interpretation of our results and keep the interpretation associational.

References S1

1. Cheng C, Spiegelman D, Li F. Estimating the natural indirect effect and the mediation proportion via the product method. *Bmc Med Res Methodol*. Nov 20 2021;21(1):253. doi:10.1186/s12874-021-01425-4

2. Health and Retirement Study. Trends and Inequalities in the Prevalence of Dementia in the United States: Replication Package. Accessed February 7, 2025. https://hrsdata.isr.umich.edu/data-products/trends-inequalities-prevalence-dementia-us-replication-package.

3. Hudomiet P, Hurd MD, Rohwedder S. Trends in inequalities in the prevalence of dementia in the United States. *P Natl Acad Sci USA*. Nov 15 2022;119(46)doi:ARTN e2212205119
10.1073/pnas.2212205119

4. Dou X, Lenzen S, Connelly LB, Lin R. Generational Differences in Age-Specific Dementia Prevalence Rates. *Jama Netw Open*. 2025;8(6):e2513384-e2513384. doi:10.1001/jamanetworkopen.2025.13384

5. Mukadam N, Wolters FJ, Walsh S, et al. Changes in prevalence and incidence of dementia and risk factors for dementia: an analysis from cohort studies. *The Lancet Public Health*. 2024;9(7):e443-e460.

6. Langa KM, Larson EB, Crimmins EM, et al. A Comparison of the Prevalence of Dementia in the United States in 2000 and 2012. *Jama Intern Med*. Jan 1 2017;177(1):51-58. doi:10.1001/jamainternmed.2016.6807

7. Larson EB, Yaffe K, Langa KM. New insights into the dementia epidemic. *N Engl J Med*. Dec 12 2013;369(24):2275-7. doi:10.1056/NEJMp1311405

8. Matthews FE, Arthur A, Barnes LE, et al. A two-decade comparison of prevalence of dementia in individuals aged 65 years and older from three geographical areas of England: results of the Cognitive Function and Ageing Study I and II. *The Lancet*. 2013;382(9902):1405-1412.

9. Imai K, Keele L, Tingley D. A General Approach to Causal Mediation Analysis. *Psychol Methods*. Dec 2010;15(4):309-334. doi:10.1037/a0020761

10. Tingley D, Yamamoto T, Hirose K, Keele L, Imai K. mediation: R Package for Causal Mediation Analysis. *J Stat Softw*. Aug 2014;59(5)
